# Acid-sensing ion channel 1a in the central nucleus of the amygdala regulates anxiety-like behaviors in a mouse model of acute pain

**DOI:** 10.3389/fnmol.2022.1006125

**Published:** 2023-01-12

**Authors:** Pei Shi, Ming-Jun Zhang, An Liu, Chen-Ling Yang, Jia-Yin Yue, Rui Hu, Yu Mao, Zhi Zhang, Wei Wang, Yan Jin, Li-Shuang Liang

**Affiliations:** ^1^Department of Anesthesiology, Linyi People's Hospital, Shandong University, Jinan, China; ^2^Stroke Center and Department of Neurology, The First Affiliated Hospital of USTC, Division of Life Sciences and Medicine, University of Science and Technology of China, Hefei, China; ^3^Department of Physiology, School of Basic Medical Sciences, Anhui Medical University, Hefei, China; ^4^Department of Endocrinology and Laboratory for Diabetes, The First Affiliated Hospital of USTC, Division of Life Sciences and Medicine, University of Science and Technology of China, Hefei, China; ^5^Department of Anesthesiology, The Third Affiliated Hospital of Anhui Medical University (The First People’s Hospital of Hefei), Hefei, China; ^6^Department of Anesthesiology and Pain Medicine, The First Affiliated Hospital of USTC, Division of Life Sciences and Medicine, University of Science and Technology of China, Hefei, China; ^7^Department of Pain, Qi lu Hospital of Shandong University, Jinan, China

**Keywords:** anxiety-like behaviors in pain, ASIC1a, GABAergic neurons, central nucleus amygdala, chemogenetic, electrophysiological recording

## Abstract

Pain is commonly comorbid with anxiety; however, the neural and molecular mechanisms underlying the comorbid anxiety symptoms in pain (CASP) have not been fully elucidated. In this study, we explored the role of acid-sensing ion channel 1a (ASIC1a), located in GABAergic neurons from the central nucleus of the amygdala (GABA^CeA^), in the regulation of CASP in an acute pain mouse model. We found that the mice displayed significant mechanical pain sensitization and anxiety-like behaviors one day post injection of complete Freud’s adjuvant (CFA1D). Electrophysiological recordings from acute brain slices showed that the activity of GABA^CeA^ neurons increased in the CFA1D mice compared with that in the saline mice. In addition, chemogenetic inhibition of GABA^CeA^ neurons relieved mechanical pain sensitization and anxiety-like behaviors in the CFA1D mice. Interestingly, through pharmacological inhibition and genetic knockdown of ASIC1a in the central nucleus amygdala, we found that downregulation of ASIC1a relieved the hypersensitization of mechanical stimuli and alleviated anxiety-related behaviors, accompanied with reversing the hyperactivity of GABA^CeA^ neurons in the CFA 1D mice. In conclusion, our results provide novel insights that ASIC1a in GABA^CeA^ neurons regulates anxiety-like behaviors in a mouse model of acute pain.

## 1. Introduction

Pain is a debilitating disease associated with many physiological and psychological maladaptations, including anxiety (Alba-Delgado et al., 2013; Simons et al., 2014; Jarrin and Finn, 2019), which may lead to prolonged pain duration and increased pain intensity (Bair et al., 2003; Zhuo, 2016). The link between pain and anxiety has proven to be increasingly significant, and both act as risk factors for each other. Multiple lines of evidence have shown that clinically, patients suffering from pain also experience anxiety (Michaelides and Zis, 2019; Tribo et al., 2020). Proper clinical treatment for comorbid anxiety symptoms in pain (CASP) is a significant challenge since the mechanism is not fully understood.

Several brain regions, such as the prefrontal cortex, anterior cingulate cortex (ACC), and amygdala, have been implicated in the central regulation of pain and emotion (Etkin et al., 2011; Barthas et al., 2015; Bliss et al., 2016). The amygdaloid complex has been well recognized in pain, reward, depression, and anxiety (Tovote et al., 2015; Wilson et al., 2019; Whittle et al., 2021); in particular, the central nucleus of the amygdala (CeA), which is well known as the “nociceptive amygdala” (Neugebauer et al., 2004; Sugimoto et al., 2021) is the main output nucleus for amygdala functions (Janak and Tye, 2015). Functional imaging studies in animals and humans displayed obvious alterations in the activity of the CeA during a painful episode (Tracey and Mantyh, 2007). These findings have led investigators to hypothesize that the CeA could be a convergent site of pain and anxiety (Senn et al., 2014). Given that the CeA consists of approximately 95% GABA neurons (Haubensak et al., 2010; Kim et al., 2017), we further investigated the role of GABAergic neurons in the CeA (GABA^CeA^) in CASP. However, little is known about how the GABA^CeA^ neurons influence and modify the molecular mechanisms of CASP.

Ion channels are emerging as ideal targets for the design and development of novel analgesics with favorable therapeutic effects (Waxman and Zamponi, 2014; Zamponi, 2016). The acid-sensing ion channel (ASIC) family consists of six subtypes (ASIC1a, 1b, 2a, 2b, 3, and 4) encoded by four genes (Hamann et al., 2015; Zhu et al., 2022). ASIC1a has been frequently studied because of its abundant levels in the mammalian central nervous system (Wemmie et al., 2003; Li et al., 2020). In addition, ASIC1a is present in areas of brain regions that are important in the pain process, including the ACC and hippocampus, as well as the amygdala (Alvarez de la Rosa et al., 2003). Furthermore, some circumstantial evidence has shown that ASIC1a is associated with specific physiological functions including aversive fear conditioning, depression, stress, anger, and some learning and memory processes (Coryell et al., 2009; Kreple et al., 2014; Yu et al., 2018; Uchitel et al., 2019). These findings suggest that ASIC1a plays an important role in the regulation of pain and anxiety-like behaviors.

In this study, we characterized the role of ASIC1a in GABA^CeA^ neurons for CASP in an acute pain mouse model. We found that pharmacological inhibition or genetic knockdown of ASIC1a in the CeA significantly attenuated mechanical hypersensitivity and anxiety-like behaviors in CFA1D mice.

## 2. Materials and methods

### 2.1. Animals and inflammatory pain model

C57BL/6 J (Stock #:000064), the *ROSA26^Ai9^* Cre-dependent tdTomato reporter (*Ai9*, Stock #:007909) and *SOM-ires-Cre* (Stock #:018973) male mice were purchased from the Charles River or Jackson Laboratories. Meanwhile, *GAD2-ires-Cre* (Stock #:028867) *or PKCδ-Cre* (GENSAT – founder line 011559-UCD) mice were crossed with *Ai9* mice to produce transgenic mice with cell type-specific red tdTomato-expressing GABA (*GAD2-tdTOM*) neurons or *PKCδ* (*PKCδ-tdTOM*) neurons.

Animal care and the experimental protocols were approved by the Animal Ethics Committee of the University of Science and Technology of China (Hefei, China). In the experiment, C57BL/6 J (Stock #:000064), the *ROSA26^Ai9^* Cre-dependent tdTomato reporter (*Ai9*, Stock #:007909) and *SOM-ires-Cre* (Stock #:018973) male mice were purchased from the Charles River or Jackson Laboratories. All mice at 2 to 3 months of age were used for these studies. *GAD2-ires-Cre* (Stock #:028867) *or PKCδ-Cre* (GENSAT, Stock #: 011559-UCD) mice were crossed with *Ai9* mice to produce transgenic mice with cell type-specific red tdTomato-expressing GABA (*GAD2-tdTOM*) neurons or *PKCδ* (*PKCδ-tdTOM*) neurons. Five animals were housed per cage at a stable temperature (23–25°C) except during cannula surgery. They were provided free access to water and food under a 12-h light/dark cycle. Acute inflammatory pain was induced by injection of CFA (10 μl, Sigma-Aldrich, MO, United States) or saline into the left hind paw with an insulin syringe (BD Corp 0.3 ml). Mice were anesthetized with 3% isoflurane (R510-22-16, Shenzhen RWD Life Science Co., Ltd., China) inhalation using an anesthesia machine (R540IP, Shenzhen RWD Life Science Co., Ltd., China) during plantar CFA injection. To avoid von Frey stimuli-induced stress effects on mice, we used different batches of mice for pain sensitivity and anxiety-like behavioral tests throughout the study.

### 2.2. von Frey filament test

Mechanical pain threshold was measured using von Frey filaments as we previously reported (Zhang et al., 2011). All the animals were subjected to the von Frey test to check their basal mechanical pain threshold before the experiment. Mice were acclimated to the testing environment and test apparatus for 3 d before the pain test. Then, the mice were individually placed in a transparent plastic box with mesh floor. The plantar surface of the hind paw was stimulated using Von Frey filaments in sequential order from 0.02 to 1.0 g. A sharp movement of the hind paw (withdrawal or licking) was considered a positive response. The threshold was measured at 5-min intervals.

### 2.3. Assessment of anxiety-like behaviors

Anxiety-like behavioral tests were conducted as we previously reported (Zhou et al., 2019). Mice were habituated to the testing environment for 2 h before the behavioral test. We used a video tracking system (EthoVision XT, Noldus, Netherlands) to record the movement trajectories of mice for 5 min, and subsequently analyzed offline.

#### 2.3.1. Open field test

The apparatus (50 × 50 × 60 cm) was actually divided into a central field (25 × 25 cm) and a periphery area. The mice were placed in the center of the instrument and allowed to move freely for 5 min. Reduced entries and time spent in the central area were regarded as indicators of anxiety-like behaviors. We cleaned the instrument with 75% ethanol after each test to eliminate olfactory cues.

#### 2.3.2. Elevated plus maze test

The elevated plus maze with two opened arms (30 × 6 cm) and two closed arms (30 × 6 × 20 cm) was elevated 100 cm above the floor. At the beginning of the test, the mice were placed on a central platform facing the open arm and allowed to explore the maze. The movement of the animals, the number of entries, and the amount of time spent in the open arms were recorded and calculated offline. Reduced entries and time spent in the open arms were regarded as indicators of anxiety-like behaviors. We cleaned the instrument with 75% ethanol after each test to eliminate olfactory cues.

### 2.4. Cannula implantation and drug microinjection

Cannulation and microinjection were performed as we previously reported (Zhu et al., 2019; Zhou et al., 2021). The experimental animals were placed in a stereotaxic instrument (RWD, Shenzhen, China) under deep anesthesia. Guide cannulas (outer diameter, 0.5 mm O.D.0.41 mm-27G/M3.5, Shenzhen RWD Life Science Co., Ltd., China) were inserted bilaterally into the CeA (AP, −1.00 mm from bregma; DV, −4.00 mm from the dura; ML, ±2.85 mm from the midline), and positioned in place with skull screws and dental cement. Animals were allowed to recover from the implantation surgery for 7 days before experimental manipulations. The mice were anesthetized during the infusion, and the drug PcTx1 (15 μM in ACSF, 250 nl per side, Peptide Institute, Osaka, Japan) was administered through a polyethylene tubing between the injector and control infusion pump. The dose of PcTx1 was chosen based on the previous literature using a similar pharmacological strategy to study the function of ASIC1a (Buta et al., 2015; Li et al., 2016; Wang et al., 2018), as well as our other preliminary experiments and calculations on the diffusion range of the drug in the CeA according to the estimated average volume of this brain region in an adult mouse. All drugs were dissolved in standard artificial cerebrospinal fluid (ACSF) and infused at a rate of 50 nl/min. ACSF was infused as a control. After drug infusion, the needle was left in place for 5 min, and experimental tests were carried out after the drug had diffused and acted on its targets. Anxiety-like behaviors were measured 30 min after drug administration. At the end of the experiment, we sliced the brain to verify the injection site. Animals with incorrect diffusion scopes were excluded from the data analysis.

### 2.5. Virus injection

Animals were placed in a stereotaxic frame (RWD, Shenzhen, China) under deep anesthesia. Throughout the experiment, we used heating pads to keep the animal’s body temperature at 36°C. A 10 μl syringe with calibrated glass micropipette was used to inject the virus into the CeA (AP, −1.00 mm; DV, −4.20 mm; ML, ±2.85 mm) at a rate of 30 nl/min driven by an infusion pump (micro4, WPI, United States). The calibrated glass micropipette was indwelled for 10 min to allow virus to diffuse in the injection site at the end of infusion.

For visualization of the SOM^CeA^ neurons, Cre-dependent rAAV-DIO-mCherry-WPRE-hGH-pA (AAV-DIO-mCherry, AAV2/8, 8.93 × 10^12^ vg/ml, 200 nl) was injected into the CeA of *SOM-Cre* mice. Viruses were packaged by BrainVTA (Wuhan, China).

The rAAV-VGAT1-hM4D(Gi)-mCherry-WPRE-hGH-pA (AAV-VGAT1-hM4Di-mCherry, AAV2/8, 2.44 × 10^12^ vg/ml, 200 nl) virus was infused bilaterally into the CeA for chemogenetic manipulation in C57 mice. The rAAV-VGAT1-mCherry-WPRE-hGH-pA (AAV-VGAT1-mCherry, AAV2/8, 2.63 × 10^12^ vg/ml, 200 nl) virus was used as the control. Three weeks after viral injection, CNO (5 mg/kg, Sigma-Aldrich, MO, United States) was intraperitoneally injected 30 min before the behavior tests. Both viruses were packaged by BrainVTA (Wuhan, China).

For gene knockdown manipulation, the virus pAAV-U6-ASIC1a-shRNA-CAG-GFP (AAV-ASIC1a-shRNA, AAV2/9, 1.49 × 10^13^ vg/ml, 200 nl) was injected bilaterally to block the expression of ASIC1a in the CeA. The pAAV-U6-negative-control-CAG-GFP (AAV-NC-Ctrl, AAV2/9, 2.16 × 10^13^ vg/ml, 200 nl) virus was used as the controls, Sunbio Medical Biotechnology (Shanghai, China) packaged the viruses.

Three weeks after viral injection, mice were anesthetized with intraperitoneal injection of pentobarbital (20 mg/kg) and transcranially perfused with ice-cold saline followed by 4% paraformaldehyde (PFA). Images of virus expression were acquired using a Zeiss LSM 880 confocal microscope. Mice with missed targets were excluded from data analysis.

### 2.6. Whole-cell patch-clamp recordings

*Brain slice preparation*. Mice were deeply anesthetized with pentobarbital sodium (2%, w/v, i.p.) and after intracardial perfusion with ~20 ml ice-cold NMDG ACSF contained the following (in mM): 93 NMDG, 25 glucose, 2.5 KCl, 2 thiourea, 20 HEPES buffer, 1.2 NaH_2_PO_4_, 30 NaHCO_3_, 0.5 CaCl_2_, 10 MgSO_4_, 5 sodium ascorbate, 3 sodium pyruvate, and 3 glutathione (pH 7.3–7.4, osmolarity of 300–305 mOsm per kg), Coronal slices (300 μm) of the CeA were sectioned with a vibrating microtome (VT1200s, Leica, Wetzlar, Germany), and the slices were incubated in an oxygenated HEPES solution (28°C) that contained (in mM) 30 NaHCO_3_, 2.5 KCl, 1.2 NaH_2_PO_4_, 20 HEPES, 2 thiourea, 25 glucose, 5 Na-ascorbate, 92 NaCl, 3 Na-pyruvate, and 2 MgSO_4_, 2 CaCl_2_, and 3 GSH (pH:7.3–7.4, osmolarity: 300–310 mOsm/kg) for at least 1 h. The brain slices were transferred to a slice chamber (Warner Instruments, United States) with ACSF that contained (in mM) 129 NaCl, 20 NaHCO_3_, 2.4 CaCl_2_, 3 KCl, 1.2 KH_2_PO_4_, 1.3 MgSO_4_, and 10 glucose (pH 7.3–7.4, osmolarity 300–305 mOsm per kg) at 2.5–3 ml/min at 32°C. The temperature of the ACSF was maintained by an in-line solution heater (TC-344B, Warner Instruments, United States).

*Whole-cell patch-clamp recording*. *In vitro* electrophysiological recordings were performed as described previously (Jin et al., 2020). Neurons in the CeA were visualized with a water immersion objective (×40) in an infrared-differential interference contrast microscope (BX51Wl, Olympus). A MultiClamp 700B amplifier and pCLAMP10.7 software were applied to collect electrophysiological signals. After a stable Gigaseal was formed, the capacitance and series resistance were automatically compensated. Current-evoked firings and tonic membrane currents (Δ*I*_m_) in GABA^CeA^ neurons were recorded under current-clamp mode (*I* = 0 pA) and under voltage-clamp mode (*V*_H_ *=* −70 mV), respectively. The pipettes (5–8 MΩ) were filled with potassium-gluconate-based internal solution, containing (in mM) 130 K-gluconate, 5 KCl, 2 MgCl_2_, 10 HEPES, 0.6 EGTA, 0.3 Na-GTP and 2 Mg-ATP (pH 7.2, osmolality of 285–290 mOsm). The recordings were made at least 5 min after establishing a whole-cell configuration with a stable resting membrane potential. The rheobase of the current-evoked action potential was defined as the minimum current needed to elicit a single action potential.

### 2.7. Immunohistochemistry

The protocol was similar to that explained previously (Yin et al., 2020). The mice were anesthetized and consecutively intracardially perfused with ice-cold saline for 3 min and 4% PFA for 4 min. Brain tissue was extracted and post-fixed overnight in 4% PFA at 4°C, and then immersed in 30% (W/V) sucrose at 4°C until they settled. Frozen brains were sectioned into transverse slices (40 μm) with a cryostat (Leica CM1860, Wetzlar, Germany). Brain slices were used for immunofluorescence. The slices were incubated in primary antibodies, including anti-ASIC1a (1:500, mouse, Abcam, Cambridge, United Kingdom), anti-PKCδ (1:250, rabbit, Abcam, Cambridge, United Kingdom), anti-SOM (1:100, mouse, Santa Cruz, Texas, United States) and anti-GABA (1:500, rabbit, Sigma, MO, United States), at 4°C for 24 h. Then, the slices were washed with PBS three times (5 min each time) and incubated with the secondary antibodies (1:500, Invitrogen, MA, United States) for 2 h in a dark place at room temperature. The slices were observed with a Zeiss LSM880 microscope (Oberkochen, Germany) to visualize the fluorescence signals.

### 2.8. Western blotting

Bilateral CeA tissue was extracted from the CFA 1D mice, and a vibratome was used to obtain 300-mm-thick sections. Membrane protein was extracted using a membrane and cytoplasmic extraction kit (Sangon Biotech, Shanghai, China, C510005) following manufacturer’s instructions. The tissue was homogenized using an automatic sample rapid grinder several times in ice-cold RIPA buffer (containing 50 mM Tris–HCl, pH 7.6, 150 mM NaCl, 1% Triton X-100, 0.1% SDS, 0.5% sodium deoxycholate, and a protease inhibitor cocktail) and then incubated on ice for 10 min. The mixture was centrifuged for 10 min at 12000 rpm and 4°C, whereupon the supernatant was extracted. The supernatant was diluted using an SDS-PAGE loading buffer. A 10% SDS-PAGE gel and 20 μg of proteins were used for electrophoresis, and then transferred to 0.22-μm polyvinylidene fluoride. After blocking with 5% nonfat milk, the membranes were incubated with primary antibodies, namely, ASIC1a (1:1000, Abcam, Cambridge, United Kingdom), β-actin (1:1000, Absin, Shanghai, China) and Na,K-ATPase (1:1000, Cell Signaling, Massachusetts, United States), at 4°C overnight and a secondary antibody (1:5000, Thermo Scientific, MA, United States) at room temperature for 90 min. The protein bands were visualized using chemiluminescence and quantified using ImageJ software. The ASIC1a levels were normalized to the β-actin levels.

### 2.9. Statistical analysis

GraphPad Prism 7 (GraphPad Software, Inc., United States) was used for the statistical analyses and graphs. Offline analysis of the data obtained from electrophysiological recordings was conducted using Clampfit software version 10.7 (Molecular Devices, Sunnyvale, CA) and MiniAnalysis software version 6.03 (Synaptosoft Inc., United States). All data are shown as the mean ± SEM, and significance levels are indicated as * *p* < 0.05, ** *p* < 0.01 and *** *p* < 0.001. We conducted simple statistical comparisons between two groups using Student’s *t*-test. One-way ANOVA, two-way ANOVA and *post-hoc* analyses were used in analyses with multiple experimental groups.

## 3. Results

### 3.1. Increased activity of GABA^CeA^ neurons in comorbid anxiety symptoms in acute pain

**Figure 1:**
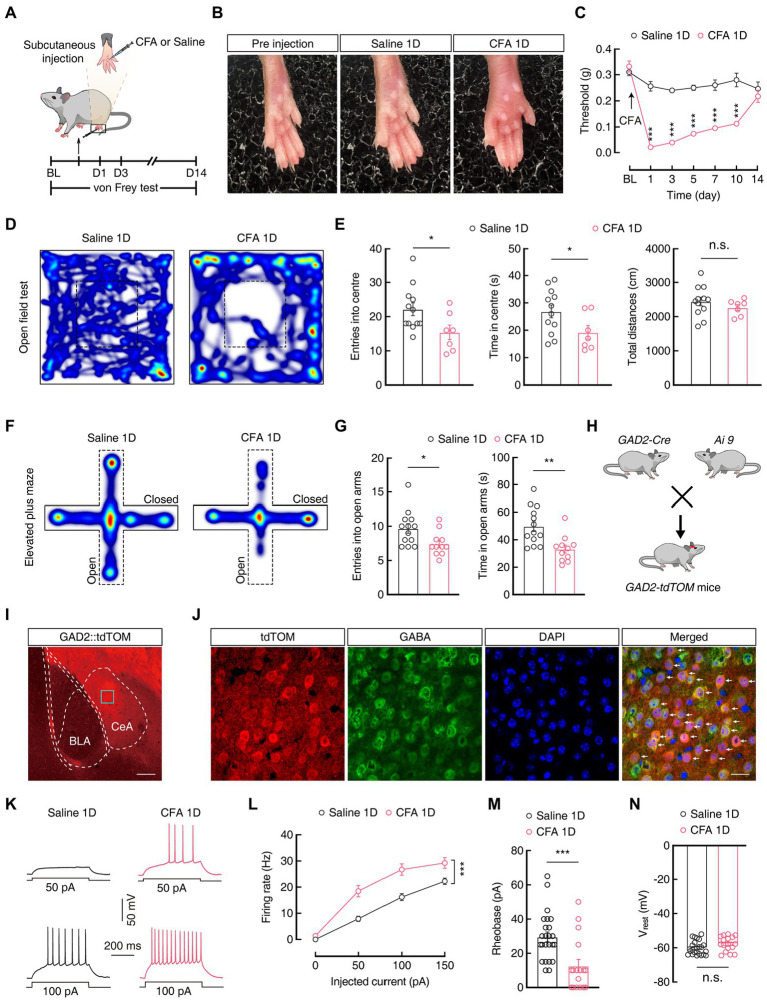
CFA 1D mice display pain and anxiety-like behaviors. **(A)** Schematic of the experimental procedure for mice injected with CFA and behavioral tests. **(B)** Left hind paw of mice on day 1 after injection of saline or CFA. **(C)** CFA-induced changes in pain threshold using the von Frey test (Saline 1D, *n* = 8 mice; CFA 1D, *n* = 7 mice; *F*_(1, 13)_ = 199.7, *p* < 0.0001). **(D,F)** Heatmaps showing locomotion traces of CFA 1D mice and Saline 1D mice in the open field test (OFT) and elevated plus maze test (EPMT). **(E,G)** Summarized data of the time spent in and entries into the central area of the OFT (**E**, Saline 1D, *n* = 12 mice; CFA 1D, *n* = 7 mice; entries into the center in the OFT, *t*_17_ = 2.282, *p* = 0.0357; time in the center in the OFT, *t*_17_ = 2.122, *p* = 0.0488; total distance, *t*_17_ = 0.9713, *p* = 0.3450) and open arms of the EPMT (**G**, Saline 1D, *n* = 13 mice; CFA 1D, *n* = 11 mice; entries into the open arms, *t*_22_ = 2.471, *p* = 0.0217; time in the open arms, *t*_22_ = 3.375, *p* = 0.0027) in the saline 1D or CFA 1D mice. **(H)** Diagram of crossing *Gad2-Cre* mice and *Ai9* reporter mice. **(I)** Representative images of GABAergic neurons expressing tdTomato in the CeA from *GAD2-tdTOM* mice. Scale bar, 200 μm. **(J)** GABA immunofluorescence were colocalized with tdTomato-labeled neurons within the CeA. Scale bar, 20 μm. **(K,L)** Sample traces **(K)** and statistical data **(L)** for action potential firing recorded from GABA^CeA^ neurons (red cells in **J**) in CFA 1D or Saline 1D mice (*n* = 24 cells from five mice for Saline1D group; *n* = 17 cells from four mice for CFA 1D group; *F*_(1, 39)_ = 18.59, *p* = 0.0001). **(M)** Summary of the rheobases of action potentials from GABA^CeA^ neurons in Saline 1D or CFA 1D mice (*n* = 24 cells from five mice for Saline1D group; *n* = 17 cells from four mice for CFA 1D group; *t*_39_ = 3.640, *p* = 0.0008). **(N)** The resting membrane potential of the CFA 1D group increased compared with that of the Saline group (*n* = 24 cells from five mice for the saline group; *n* = 17 cells from four mice for the CFA 1D group; *t*_39_ = 1.828, *p* = 0.0751). All data are shown as the mean ± SEM, n.s., not significant; * *p* < 0.05; ** *p* < 0.01 and *** *p* < 0.001. Two-way repeated-measures ANOVA with Bonferroni *post-hoc* analysis for **(C,L)**; unpaired *t*-test for **(E,G,M,N)**.

Anxiety-like behaviors in acute pain have been proven in animal models (Zhou et al., 2021). Here, we established an animal model of anxiety-like behaviors in acute pain 1 day after complete Freud’s adjuvant (CFA) injection (CFA 1D) into the left hind paw (Figures 1A,B). After CFA injection, the mechanical pain threshold decreased significantly compared with that of the saline group (Day 1: 0.26 ± 0.02 g *vs* 0.02 ± 0.002 g; Saline 1D, *n* = 8 mice; CFA 1D, *n* = 7 mice; Figure 1C). In addition, the anxiety-like behaviors were assessed in the mice at 1, 3, and 5 days after CFA injection using the elevated plus maze test (EPMT) and open field test (OFT). Interestingly, we found that entries and time spent in the open arms decreased in the EPMT (entries: 9.69 ± 0.70 *vs* 7.46 ± 0.53; time: 49.85 ± 3.78 s *vs* 33.31 ± 2.90 s; Saline 1D, *n* = 13 mice; CFA 1D, *n* = 11 mice; Figures 1F,G) and the center of the OFT (entries: 22.17 ± 1.88 *vs* 15.43 ± 2.11; time: 26.91 ± 2.34 s *vs* 19.21 ± 2.50 s; Saline 1D, *n* = 12 mice; CFA 1D, *n* = 7 mice; Figures 1D,E) in the CFA1D mice compared with those in the Saline 1D mice. However, the acute pain-induced anxiety-like behaviors observed in CFA 1D mice disappeared in CFA 3D and CFA 5D mice (Supplementary Figure S1). In addition, total travel distance did not change in the CFA group compared with that of the control group (Figure 1E), suggesting that exercise capacity was not affected by the CFA injection dose in this study. These results suggest that anxiety-like behaviors were reliably induced by the current acute inflammatory pain model.

Next, we investigated the activity of GABA^CeA^ neurons using whole-cell patch-clamp recordings. To visualize GABA neurons, *glutamic acid decarboxylase 2* (GAD2, a GABA synthetic enzyme)*-Cre* mice were crossed with *Ai9* (RCL-tdT) mice (Madisen et al., 2010) to produce transgenic mice with cell-type-specific red tdTomato^+^ GABA (*GAD2-tdTOM*) neurons (Figure 1H). Immunofluorescence staining showed that the tdTomato-labeled neurons in the CeA were colocalized with the GABA-specific antibody (Figures 1I,J), which is consistent with previous research, suggesting that the CeA contains 95% GABA neurons (Haubensak et al., 2010; Janak and Tye, 2015; Martin-Fernandez et al., 2017). Under current-clamp recordings in acute brain slices on GABA^CeA^ neurons, we found an increase in the firing rate and a decrease in the rheobase of current-elicited action potentials (Firing rate: 100 pA 16.19 ± 1.33 *vs* 26.71 ± 2.19; Rheobase: 29.58 ± 2.87 pA *vs* 12.65 ± 3.77 pA; Saline 1D, *n* = 24 cells from five mice; CFA 1D, *n* = 17 cells from four mice; Figures 1K–M), with no change in the resting membrane potential (V_rest_; Figure 1N) in the CFA 1D mice compared with those in the saline group. These results indicate an increase in the excitability of GABA^CeA^ neurons in the CFA1D mice.

### 3.2. The excitability of PKCδ^CeA^ and SOM^CeA^ neurons is increased in CFA 1D mice

**Figure 2:**
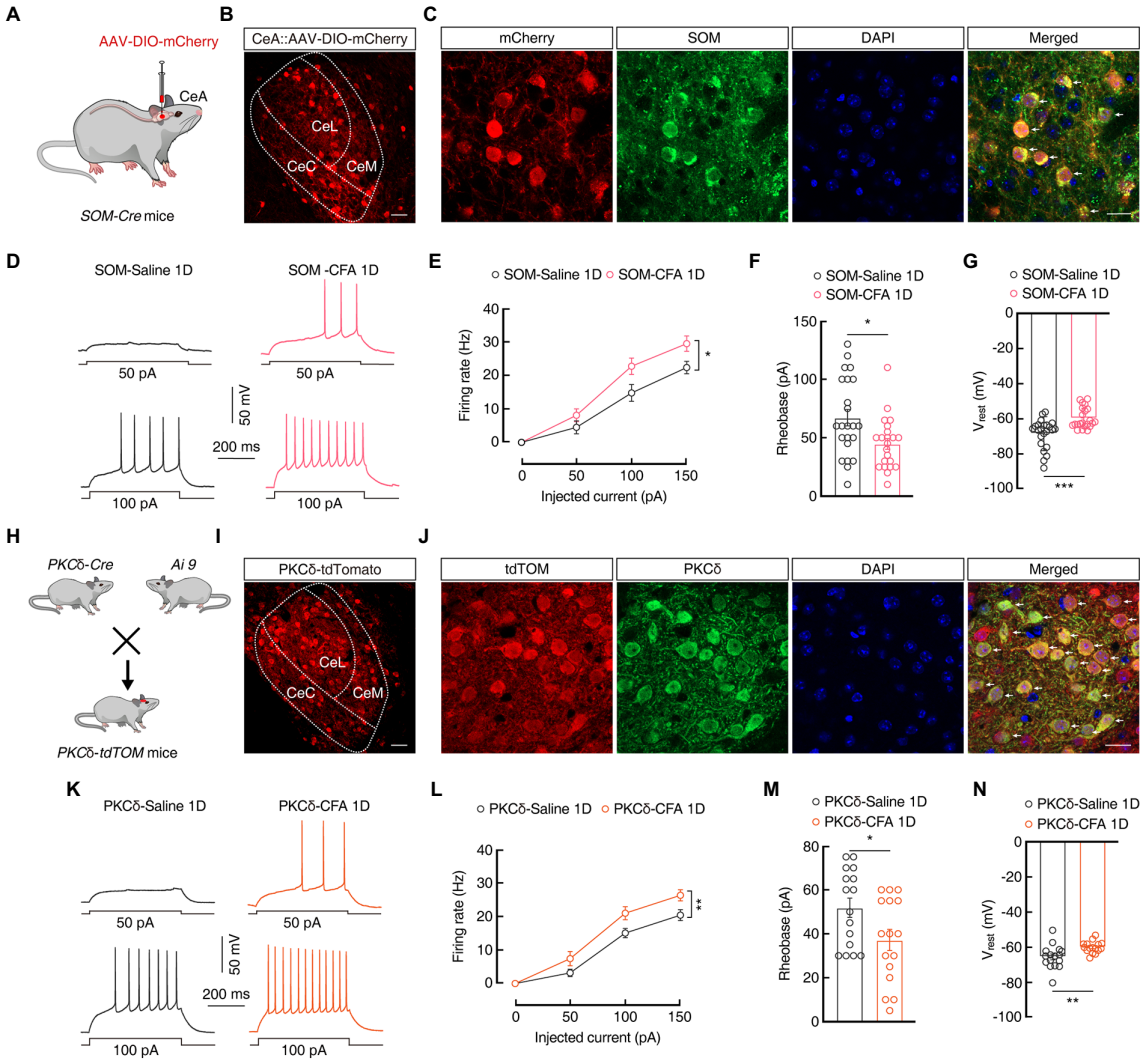
The excitability of PKCδ^CeA^ and SOM^CeA^ neurons is increased in CFA 1D mice. **(A)** Schematic of AAV-DIO-mCherry injected into the *SOM-Cre* mice. **(B)** Representative images of AAV-DIO-mCherry expression in the *SOM-Cre* mice. Scale bar, 40 μm. **(C)** The mCherry-labeled neurons within the CeA were colocalized with the SOM immunofluorescence. Scale bar, 20 μm. **(D,E)** Sample traces **(D)** and statistical data **(E)** for action potential firing recorded from SOM^CeA^ neurons (red cells in **C**) in SOM-CFA 1D or SOM-Saline 1D mice (*n* = 23 cells from five mice for SOM-Saline 1D group; *n* = 22 cells from five mice for SOM-CFA 1D group; *F*_(1, 43)_ = 4.807, *p* = 0.0338). **(F)** Summary of the rheobases of action potentials from SOM^CeA^ neurons (red cells in **C**) in SOM-CFA 1D or SOM-Saline 1D mice (*n* = 23 cells from five mice for SOM-Saline 1D group; *n* = 22 cells from five mice for SOM-CFA 1D group; *t*_43_ = 2.603, *p* = 0.0126). **(G)** The resting membrane potential (V_rest_) of the SOM-CFA 1D group increased compared with that of the saline group (*n* = 23 cells from five mice for the SOM-Saline 1D group; *n* = 22 cells from five mice for the SOM-CFA 1D group; *t*_43_ = 4.129, *p* = 0.0002). **(H)** Diagram of crossing *PKCδ-Cre* mice and *Ai9* reporter mice. **(I)** Representative images of PKCδ neurons expressing tdTomato in the CeA from *PKCδ-tdTOM* mice. Scale bar, 40 μm. **(J)** PKCδ immunofluorescence were colocalized with tdTomato-labeled neurons within the CeA. Scale bar, 20 μm. **(K,L)** Sample traces **(K)** and statistical data **(L)** for action potential firing recorded from PKCδ^CeA^ neurons (red cells in J) in CFA 1D or Saline 1D *PKCδ-tdTOM* mice (*n* = 16 cells from four mice for per group; *F*_(1, 30)_ = 10.22, *p* = 0.0033). **(M)** Summary of the rheobases of action potentials from PKCδ^CeA^ neurons (red cells in **J**) in CFA 1D or Saline 1D *PKCδ-tdTOM* mice (*n* = 16 cells from four mice for per group; *t*_30_ = 2.254, *p* = 0.0317). **(N)** The resting membrane potential (V_rest_) of the PKCδ-CFA 1D group increased compared with that of the saline group (*n* = 16 cells from four mice for per group; *t*_30_ = 2.973, *p* = 0.0058). All data are shown as the mean ± SEM, * *p* < 0.05; ** *p* < 0.01 and *** *p* < 0.001. Two-way repeated-z measures ANOVA with Bonferroni *post-hoc* analysis for **(E,L)**; unpaired *t*-test for **(F,G,M,N)**.

To test whether the regulation of pain and anxiety-like behaviors in the CeA is cell type-specific, we focused our research on two genetically identified cell types, SOM^CeA^ and PKCδ^CeA^, as these constitute the majority of CeA neurons and represent largely non-overlapping populations (Li et al., 2013; Kim et al., 2017). To visualization of SOM^CeA^ neurons, AAV-DIO-mCherry virus were injected into the CeA of *SOM-Cre* mice (Figures 2A,B) and immunofluorescence staining showed that mCherry^+^ SOM^CeA^ neurons were co-localized with the SOM-specific antibody (Figure 2C). In addition, under whole-cell recordings in the acute brain slices, we found an increase in the firing rate, a decrease in the rheobase of action potentials (Firing rate: 100 pA 14.75 ± 2.53 *vs* 22.74 ± 2.46; Rheobase: 67.17 ± 6.99 pA *vs* 45 ± 4.75 pA; SOM-Saline 1D, n = 23 cells from five mice; SOM-CFA 1D, *n* = 22 cells from five mice; Figures 2D–F), and a depolarization of the V_rest_ (−68.39 ± 1.71 mV *vs-59*.59 ± 1.25 mV, SOM-Saline 1D, *n* = 23 cells from five mice; SOM-CFA 1D, *n* = 22 cells from five mice; Figure 2G) in the mCherry^+^ SOM^CeA^ neurons from CFA 1D mice, compared with those in the Saline 1D group. These results indicated that the excitability of SOM^CeA^ neurons was increased in the CFA-induced acute pain mice.

Further, we investigated the activity of PKCδ^CeA^ neurons in transgenic mice with cell type-specific red tdTomato^+^ PKCδ^CeA^ neurons (Figures 2H,I), which were co-localized with the PKCδ-specific antibody (Figure 2J). Of note, the excitability of PKCδ^CeA^ neurons was also increased in the tdTomato^+^ PKCδ^CeA^ neurons from CFA 1D mice, compare with that in Saline 1D group (Firing rate: 100 PA, 15.08 ± 1.38 *vs* 22.23 ± 1.56; Rheobase: 51.88 ± 4.38 pA *vs* 37.19 ± 4.83 pA; *n* = 16 cells from four mice per group; V_rest_: −65.47 ± 1.66 mV *vs-60* ± 0.80 mV, *n* = 16 cells from four mice per group; Figures 2K–N). These findings likely suggest that SOM^CeA^ and PKCδ^CeA^ neurons exert synergistic effects to increase the inhibitory outputs from the CeA in the current CFA-induced inflammatory mouse model.

### 3.3. Inhibition of GABA^CeA^ neurons relieved comorbid anxiety symptoms in acute pain

**Figure 3:**
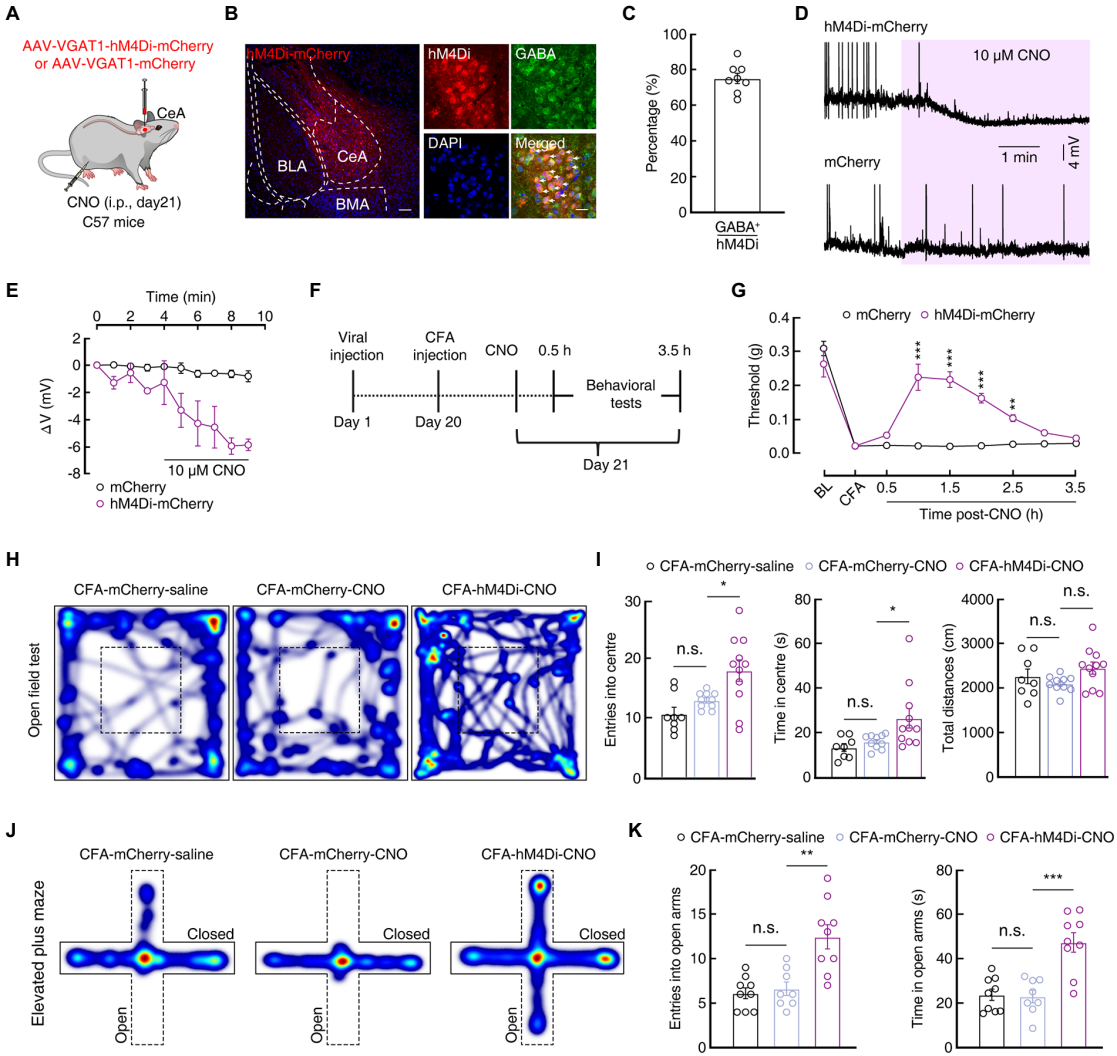
Increased GABA^CeA^ neuronal excitability is required for pain and anxiety-like behaviors. **(A)** Schematic of Chemogenetic viruses injected into the CeA of C57 mice. **(B,C)** Images **(B)** and statistics data **(C)** showing that GABA immunofluorescence were colocalized with mCherry-labeled neurons within the CeA (*n* = 6 slices from three mice). Scale bar, 20 μm. **(D,E)** Sample traces **(D)** and statistical data **(E)** of GABA^CeA^ neurons in C57 mice with CeA infusion of AAV-VGAT1-hM4Di-mCherry or AAV-VGAT1–mCherry before and after CNO intraperitoneal injection. (mCherry, *n* = 3 cells from three mice; hM4Di-mCherry, *n* = 4 cells from three mice; *F*_(1, 5)_ = 15.59, *p* = 0.0109). **(F)** Schematic diagram of the chemogenetic experimental procedure. **(G)** Chemogenetic inhibition of GABA^CeA^ neurons reversed CFA-induced mechanical hyperalgesia in the hM4Di-mCherry mice. (*n* = 7 mice per group; *F*_(1, 12)_ = 38.29, *p* < 0.0001). **(H,J)** Heatmaps showing the locomotion traces of CFA-mCherry-saline mice, CFA-mCherry-CNO mice and CFA-hM4Di-CNO mice in the open field test (OFT) and elevated plus maze test (EPMT). **(I,K)** Chemogenetic silencing of GABA^CeA^ alleviated CFA-induced anxiety-like behaviors in CFA-hM4Di-CNO mice in the OFT and EPMT (**I**, CFA-mCherry-Saline, *n* = 8 mice; CFA-mCherry-CNO, *n* = 10 mice; CFA-hM4Di-CNO, *n* = 11 mice in the OFT; entries into the center in the OFT, *F*_(2, 26)_ = 7.889, *p* = 0.0021; time in the center in the OFT, F_(2, 26)_ = 5.303, *p* = 0.0117; total distances in the OFT, F_(2, 26)_ = 2.169, *p* = 0.1346; **K**, CFA-mCherry-Saline, *n* = 9 mice; CFA-mCherry-CNO, *n* = 8 mice; CFA-hM4Di-CNO, *n* = 9 mice in EPMT; entries into the open arms in the EPMT, *F*_(2, 23)_ = 13.36, *p* = 0.0001; time in the open arms in the EPMT, F_(2, 23)_ = 16.77, *p* < 0.0001). All data are shown as the mean ± SEM, n.s., not significant; * *p* < 0.05; ** *p* < 0.01 and *** *p* < 0.001. Two-way repeated-measures ANOVA with Bonferroni *post-hoc* analysis for **(E,G)**; one-way ANOVA with Bonferroni *post-hoc* analysis for **(I,K)**.

Giving that the excitability of SOM^CeA^ and PKCδ^CeA^ neurons was increased, we therefore focused our attention on the function of GABA^CeA^ neurons in CFA-induced acute pain mice in the subsequent experiments. To further probe how GABA^CeA^ participated in anxiety-like behaviors in acute pain, we employed the chemogenetic inhibitory rAAV-VGAT1-hM4D(Gi)-mCherry-WPRE-hGH-pA (AAV-VGAT1-hM4Di-mCherry) virus under the regulation of a VGAT1 promoter and the intraperitoneal injection of its ligand clozapine-N-oxide (CNO, i.p., 5 mg/kg) to selectively inhibit GABA^CeA^ neurons in the CFA 1D mice (Figure 3A). Immunofluorescence staining showed that hM4Di-mCherry^+^ signals were mostly (~75%) colocalized with the GABA-specific antibody (Figures 3B,C). To verify the function of the chemogenetic virus, the GABA^CeA^ neurons labeled with mCherry were recorded under the current-clamp recordings from brain slices. We found that the membrane potential of the hM4Di-mCherry^+^ GABA^CeA^ neurons was significantly hyperpolarized after CNO (10 μM) administration (Figures 3D,E).

In addition, we investigated whether chemogenetic silencing of the activity of GABA^CeA^ neurons could reverse the pain and anxiety-like behaviors in the CFA 1D mice (Figure 3F). The mechanical pain threshold increased significantly in the hM4Di-mCherry group compared with that in the control group after the intraperitoneal injection of CNO (1 h: 0.02 ± 0.002 g *vs* 0.22 ± 0.04 g; *n* = 7 mice per group; Figure 3G), as well as the anxiety-like behaviors were significantly alleviated: OFT (i.e., increased time and entries into the center zone; entries: 12.90 ± 0.43 *vs* 17.91 ± 1.80; time: 15.89 ± 0.99 s *vs* 26.41 ± 4.37 s; CFA-mCherry-CNO, *n* = 10 mice; CFA-hM4Di-CNO, *n* = 11 mice; Figures 3H,I), and EPMT (i.e., increased open arms time and entries; entries: 6.63 ± 0.75 *vs* 12.44 ± 1.35; time: 23.01 ± 2.82 s *vs* 47.29 ± 4.40 s; CFA-mCherry-CNO, *n* = 8 mice; CFA-hM4Di-CNO, *n* = 9 mice; Figures 3J,K) in the CFA-hM4Di-CNO group compared with those in the control group in the CFA1D mice. Furthermore, there was no significant difference in the mechanical pain threshold and anxiety-like behaviors between hM4Di-mCherry group and control groups after CNO injection (i.p.) in Saline 1D mice (Supplementary Figure S2). Taken together, these results indicated that chemogenetic inhibition of the activity of GABA^CeA^ could relieve pain sensitization and anxiety-like behaviors in the CFA1D mice.

### 3.4. ASIC1a-mediated membrane currents are enhanced in GABA^CeA^ neurons in CFA 1D mice

**Figure 4:**
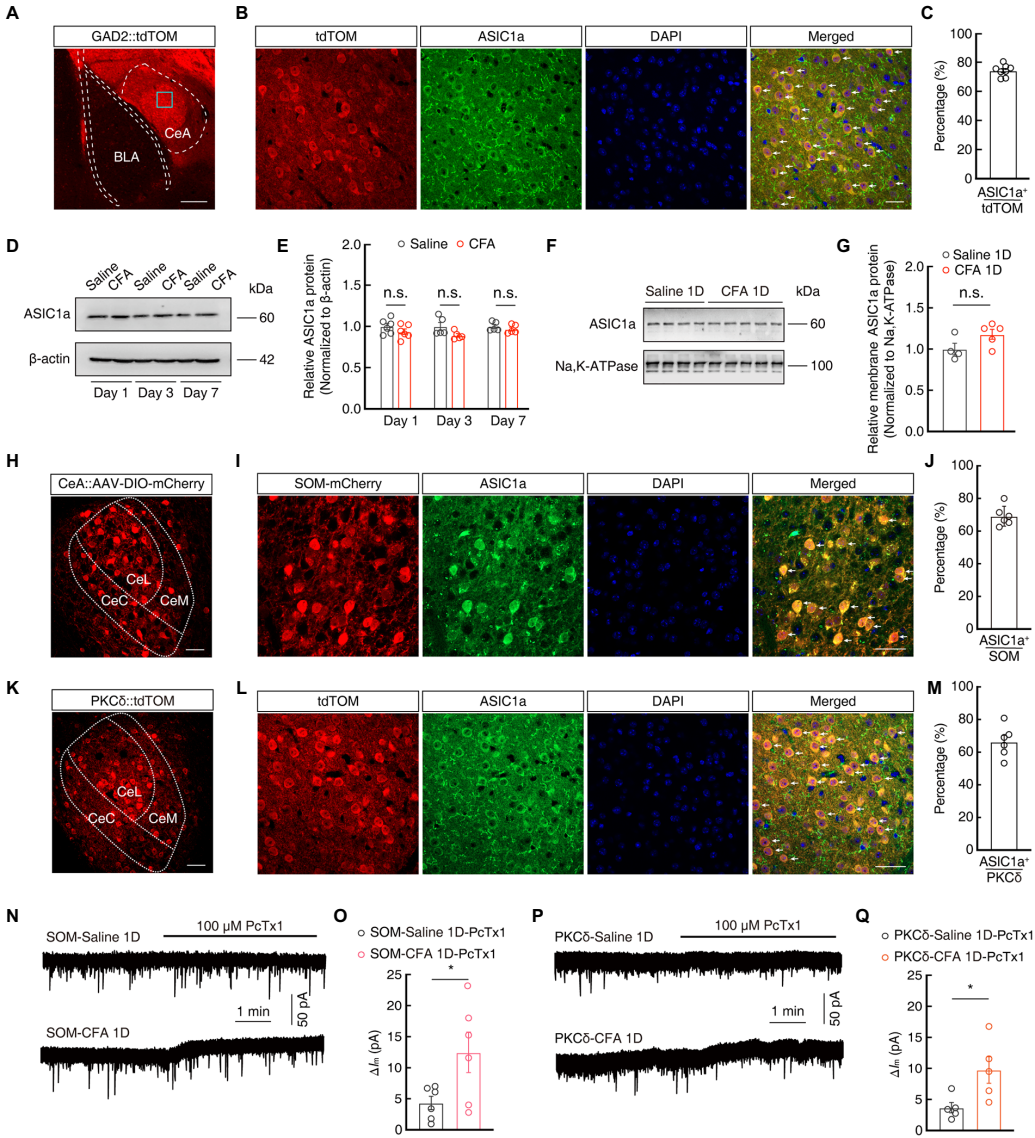
The expression and function of ASIC1a in GABA^CeA^ neurons. **(A)** Representative images of GABAergic neurons expressing tdTomato in the CeA from GAD2-tdTOM mice. Scale bars, 200 μm. **(B,C)** Images **(B)** and statistics data **(C)** showing that ASIC1a immunofluorescence were colocalized with tdTomato-labeled neurons within the CeA (*n* = 6 slices from three mice). Scale bar, 40 μm. (**D,E**) Representative images **(D)** and quantification **(E)** of Western blots showing that CFA injection did not induce significant changes in the expression of total ASIC1a in CeA (**E**, Day 1: *t*_10_ = 1.28, *p* = 0.2295, *n* = 6 mice per group; Day 3: *t*_8_ = 2.064, *p* = 0.0729, *n* = 5 mice per group; Day 7: *t*_8_ = 0.9581, *p* = 0.3661, *n* = 5 mice per group). **(F,G)** Representative images **(F)** and quantification **(G)** of Western blots showing that the membrane content of ASIC1a in CeA were not changed in mice 1 day after CFA injection, compared with Saline 1D group (**G**, Saline 1D, *n* = 4 mice; CFA 1D, *n* = 5 mice; *t*_7_ = 1.93, *p* = 0.0949). **(H)** Representative images of AAV-DIO-mCherry expression in the *SOM-Cre* mice. Scale bar, 40 μm. **(I,J)** Images **(I)** and statistics data **(J)** showing that ASIC1a immunofluorescence were colocalized with mCherry-labeled neurons within the CeA (*n* = 6 slices from three mice). Scale bar, 40 μm. **(K)** Representative images of PKCδ neurons expressing tdTomato in the CeA from PKCδ-tdTOM mice. Scale bars, 40 μm. **(L,M)** Images **(L)** and statistics data **(M)** showing that ASIC1a immunofluorescence were colocalized with tdTomato-labeled neurons within the CeA (*n* = 6 slices from three mice). Scale bar, 40 μm. **(N,O)** Representative current traces **(N)** and summarized data **(O)** showing that the application of PcTx1 (100 μM) induced larger outward currents in the SOM^CeA^ neurons in acute brain slices of CFA 1D mice (*n* = 6 cells from five mice for per group; *t*_10_ = 2.372, *p* = 0.0391). **(P,Q)** Representative current traces **(P)** and summarized data **(Q)** showing that the application of PcTx1 (100 μM) induced larger outward currents in the PKCδ^CeA^ neurons in acute brain slices of CFA 1D mice (*n* = 5 cells from five mice for per group; *t*_8_ = 2.660, *p* = 0.0288). All data are shown as the mean ± SEM, n.s., not significant; * *p* < 0.05. unpaired *t*-test for **(E,G,O,Q)**.

Previous studies have reported that ASIC1a plays an important role in pain processing in the peripheral nervous system (Bohlen et al., 2011; Diochot et al., 2012; Duan et al., 2012). We next explored the expression and function of ASIC1a in the CeA neurons in the current mouse model. Immunofluorescence staining showed that tdTomato^+^ signals in the CeA from *GAD2-tdTOM* mice were mostly (~74%) colocalized with the ASIC1a-specific antibodies (Figures 4A–C). In addition, western blotting showed that the total protein levels of ASIC1a unchanged in mice on days 1, 3, and 7 after CFA injection, compared with saline group (Figures 4D,E). In addition, the amount of ASIC1a protein on the plasma membrane prepared from the CeA was not different between Saline 1D and CFA 1D mice (Figures 4F,G). To explore whether ASIC1a is expressed on a neuron-specific type, we then performed immunofluorescence staining and found that ASIC1a was highly co-labeled with both the mCherry^+^ SOM^CeA^ neurons (~70%) and tdTomato^+^ PKCδ^CeA^ neurons (~66%; Figures 4H–M). These results suggested that, at least in SOM^CeA^ and PKCδ^CeA^ neurons in the CeA, ASIC1a expression is not neuron-specific but widespread.

To further investigate the sensitivity of ASIC1a, we next examined ASIC1a-mediated tonic currents of GABA^CeA^ neurons using whole-cell recordings. At the membrane holding potential of −70 mV, we found that the application of PcTx1 in the ACSF induced larger outward currents (Δ*I*_m_) in mCherry^+^ SOM^CeA^ neurons and tdTomato^+^ PKCδ^CeA^ neurons recorded in CFA 1D mice, compared to those of Saline 1D mice (SOM^CeA^: 4.33 ± 1.07 *vs* 12.48 ± 3.26; PKCδ^CeA^: 3.67 ± 0.83 *vs* 9.75 ± 2.13; Figures 4N–Q). Taken together, these results suggested that there was no significant variation in ASIC1a protein expression, but only an increase in its sensitivity in GABA^CeA^ neurons, in CFA-induced acute pain mice in the current study.

### 3.5. ASIC1a in the CeA regulated pain hypersensitivity and anxiety-like behaviors in the CFA1D mice

**Figure 5:**
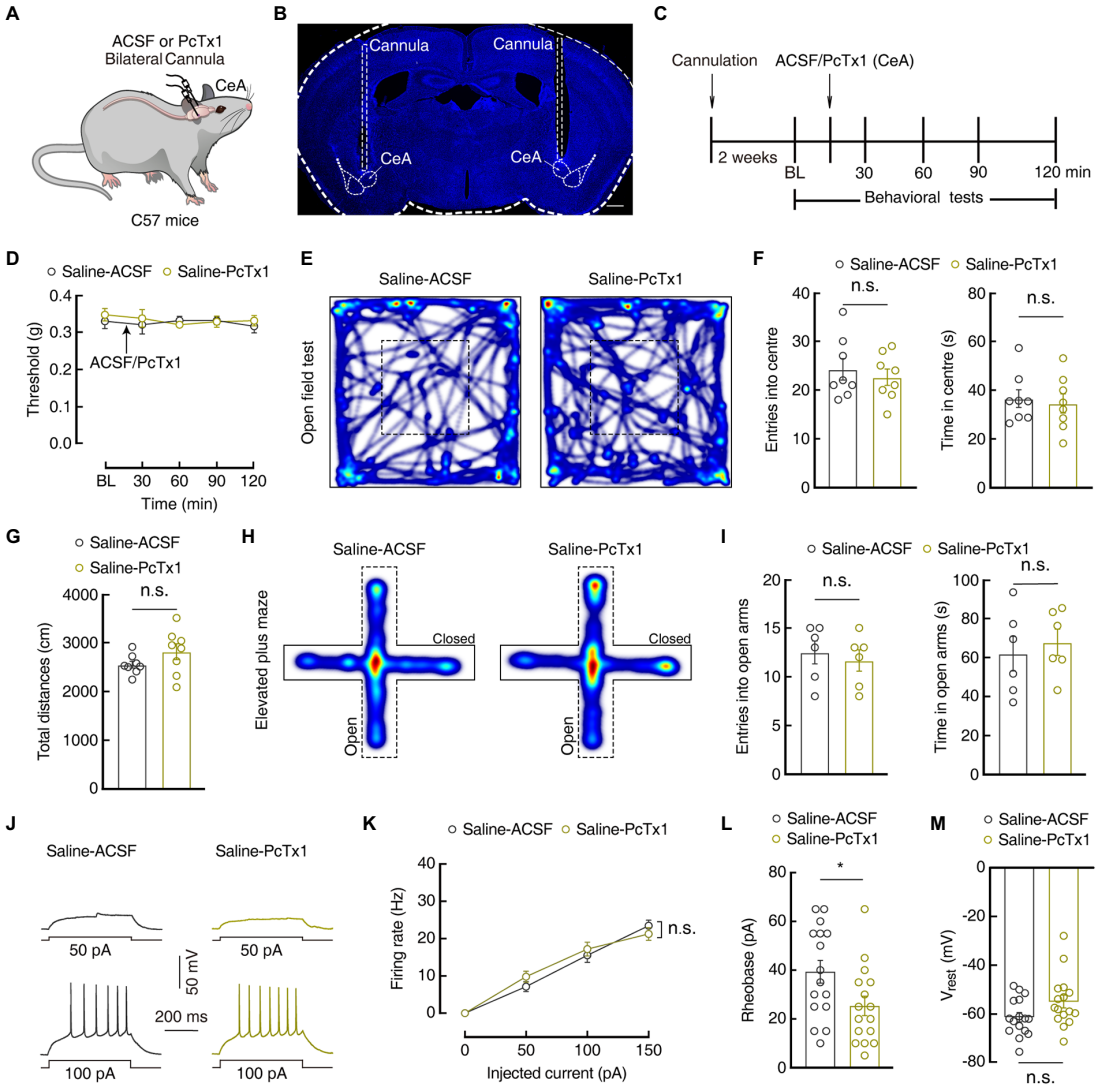
Pharmacological manipulations of ASIC1a in the CeA did not affect normal sensation and mood. **(A)** Schematic of bilateral PcTx1 injection into the CeA of C57 mice. **(B)** Typical image of bilateral cannula injection sites. Scale bar, 500 μm. **(C)** An outline of the pharmacological experimental procedure. **(D)** The mechanical pain threshold did not change after CeA injection of PcTx1 in Saline 1D mice (Saline-ACSF, *n* = 7 mice; Saline-PcTx1, *n* = 6 mice; *F*_(1,11)_ = 0.4129, *p* = 0.5337). **(E,H)** Heatmaps showing the locomotion traces of Saline-ACSF mice and Saline-PcTx1 mice in the open field test (OFT) and elevated plus maze test (EPMT). **(F,G,I)** Summarized data showing no difference between ACSF and PcTx1-treated groups in OFT and EMPT in Saline 1D mice (**F**, *n* = 8 mice per group in OFT; entries in center in OFT, *t*_14_ = 0.5869, *p* = 0.5666; time in center in OFT, *t*_14_ = 0.3806, *p* = 0.7092; G, total distances in OFT, *t*_14_ = 1.504, *p* = 0.1549; **I**, *n* = 6 mice per group in EPMT; entries in the open arms in EPMT, *t*_10_ = 0.5207, *p* = 0.6139; time in the open arms in EPMT, *t*_10_ = 0.5167, *p* = 0.6166). **(J,K)** Sample traces **(J)** and statistical data **(K)** for action potential firing recorded from GABA^CeA^ neurons in Saline 1D mice treated with ACSF or PcTx1 (*n* = 16 cells from four mice for per group; *F*_(1,30)_ = 0.1375, *p* = 0.7134). **(L)** Summary of the rheobases of action potentials from Saline 1D mice treated with ACSF or PcTx1 (*n* = 16 cells from four mice for per group; *t*_30_ = 2.294, *p* = 0.029) **(M)** The resting membrane potential (V_rest_) showing no difference between Saline-ACSF mice and Saline-PcTx1 mice (*n* = 16 cells from four mice for per group; *t*_30_ = 1.947, *p* = 0.0610). All data are shown as the mean ± SEM, n.s., not significant; * *p* < 0.05. Two-way repeated-measures ANOVA with Bonferroni *post-hoc* analysis for **(D,K)**; unpaired *t*-test for **(F,G,I,L,M)**.

Previous studies have reported that ASIC1a plays an important role in pain processing in the peripheral nervous system (Bohlen et al., 2011; Diochot et al., 2012; Duan et al., 2012). We next evaluated whether ASIC1a in GABA^CeA^ neurons also contributes to pain and anxiety-like behaviors in the current mouse model. Then we bilaterally injected PcTx1 (15 μM), a selective ASIC1a inhibitor, into the CeA (Figures 5A,B; Supplementary Figure S3). Behavioral tests showed that administration of PcTx1 in Saline 1D mice did not affect the mechanical pain threshold and anxiety-like behaviors compared with the administration of ACSF in Saline 1D mice (Figures 5C–I). In addition, compared with the ACSF-treated Saline 1D mice, whole-cell recordings in the brain slices showed that GABA^CeA^ neuronal activity was not changed in the PcTx1-treated Saline 1D mice (Figures 5J–M).

**Figure 6:**
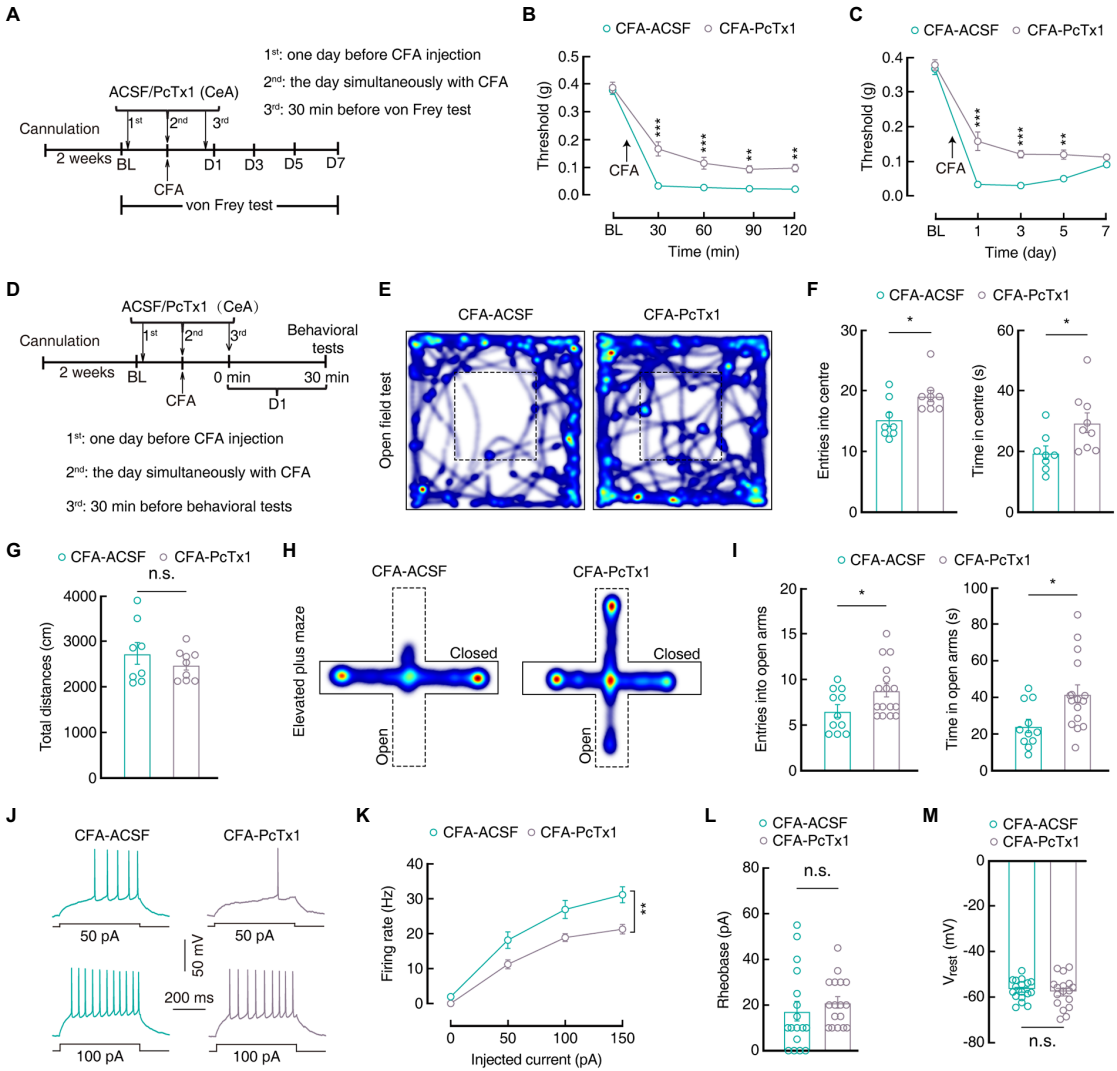
Inhibiting ASIC1a in the CeA reverses inflammatory pain hypersensitivity and anxiety-like behaviors. **(A)** Schematic diagram of the pharmacological experimental procedure. **(B)** Mechanical pain thresholds of CFA 1D mice following the CeA injection of PcTx1 for 2 h (CFA-ACSF, *n* = 7 mice; CFA-PcTx1, *n* = 7 mice; *F*_(1,12)_ = 50.97, *p* < 0.0001). **(C)** Mechanical pain thresholds of CFA 1D mice following the CeA injection of PcTx1 for 1 week (CFA-ACSF, *n* = 7 mice; CFA-PcTx1, *n* = 7 mice; F_(1,12)_ = 42.03, *p* < 0.0001). **(D)** A pharmacological experimental procedure. **(E,H)** Heatmaps showing the locomotion traces of CFA-ACSF mice and CFA-PcTx1 mice in the open field test (OFT) and elevated plus maze test (EPMT). **(F,G,I)** Behavioral effects of the pharmacological inhibition of ASIC1a in the CeA in OPT and EPMT (**F**, CFA-ACSF, *n* = 8 mice,CFA-PcTx1, *n* = 9 mice in OFT; entries into center in OFT, *t*_15_ = 2.655, *p* = 0.018; time in center in OFT, *t*_15_ = 2.436, *p* = 0.0278; **G**, total distances in OFT, *t*_15_ = 0.9944, *p* = 0.3358; **I**, CFA-ACSF, *n* = 11 mice, CFA-PcTx1, *n* = 16 mice in EPMT; entries into the open arms in EPMT, *t*_25_ = 2.174, *p* = 0.0394; time in the open arms in EPMT, *t*_25_ = 2.626, *p* = 0.0145). **(J,K)** Sample traces **(J)** and statistical data **(K)** for action potential firing recorded from GABA^CeA^ in CFA 1D mice treated with ACSF or PcTx1 (*n* = 17 cells from four mice per group; *F*_(1,32)_ = 10.66, *p* = 0.0026). **(L)** Summary of the rheobases of action potentials from CFA 1D mice treated with ACSF or PcTx1 (*n* = 17 cells from four mice per group; *t*_32_ = 0.7657, *p* = 0.4495). **(M)** The resting membrane potential (V_rest_) showing no difference between CFA-ACSF mice and CFA-PcTx1 mice (*n* = 17 cells from four mice per group; *t*_32_ = 0.5323, *p* = 0.5982). All data are shown as the mean ± SEM, n.s., not significant; * *p* < 0.05; ** *p* < 0.01 and *** *p* < 0.001. Two-way repeated-measures ANOVA with Bonferroni *post-hoc* analysis for **(B,C,K)**; unpaired *t*-test for **(F,G,I,L,M)**.

Interestingly, following the infusion of PcTx1 (Figure 6A) on 1 day before, during, and 1 day after CFA injection in mice, the mechanical pain threshold increased significantly in the CFA1D mice compared with that in the ACSF-treated CFA1D mice (Day1: 0.04 ± 0.002 g *vs* 0.16 ± 0.03 g; *n* = 7 mice per group; Figures 6B,C). Furthermore, the time spent in and the entries into the center of the OFT(entries: 15.25±1.13 *vs* 19.11± 0.93; time: 19.60 ± 2.22 s *vs* 29.44±3.25 s; CFA-ACSF, *n* = 8 mice; CFA-PcTx1, *n* = 9 mice), as well as the time spent in and the entries into the open arms of the EPMT (entries: 6.54±0.69 *vs* 8.81± 0.72; time: 24.38 ± 3.63 s *vs* 41.86±4.90 s; CFA-ACSF, *n* = 11 mice; CFA-PcTx1, *n* = 16 mice) increased in the PcTx1-treated CFA1D mice compared with those in the ACSF-treated CFA1D mice (Figures 6D–I). These data suggest that the bilateral administration of PcTx1 within the CeA significantly relieved mechanical pain sensitization and anxiety-like behaviors in the CFA 1D mice. To determine whether the pharmacological inhibition of ASIC1a affects the activity of GABA^CeA^ neurons, we performed whole-cell current-recordings in the brain slices and found that the hyperactivity of GABA^CeA^ neurons decreased (Firing rate: 100 pA, 26.94 ± 2.58 vs. 18.88 ± 1.14; *n* = 17 cells from four mice per group; Figures 6J–L), with no change in the V_rest_ (Figure 6M) in the PcTx1-treated CFA1D mice compared with the that in the ACSF-treated CFA1D mice. These results indicate that the pharmacological inhibition of ASIC1a could reverse CASP in an acute pain mouse model by suppressing the activity of GABA^CeA^ neurons.

### 3.6. Genetic disruption of ASIC1a alleviated pain hypersensitivity and anxiety-like behaviors in the CFA1D mice

**Figure 7:**
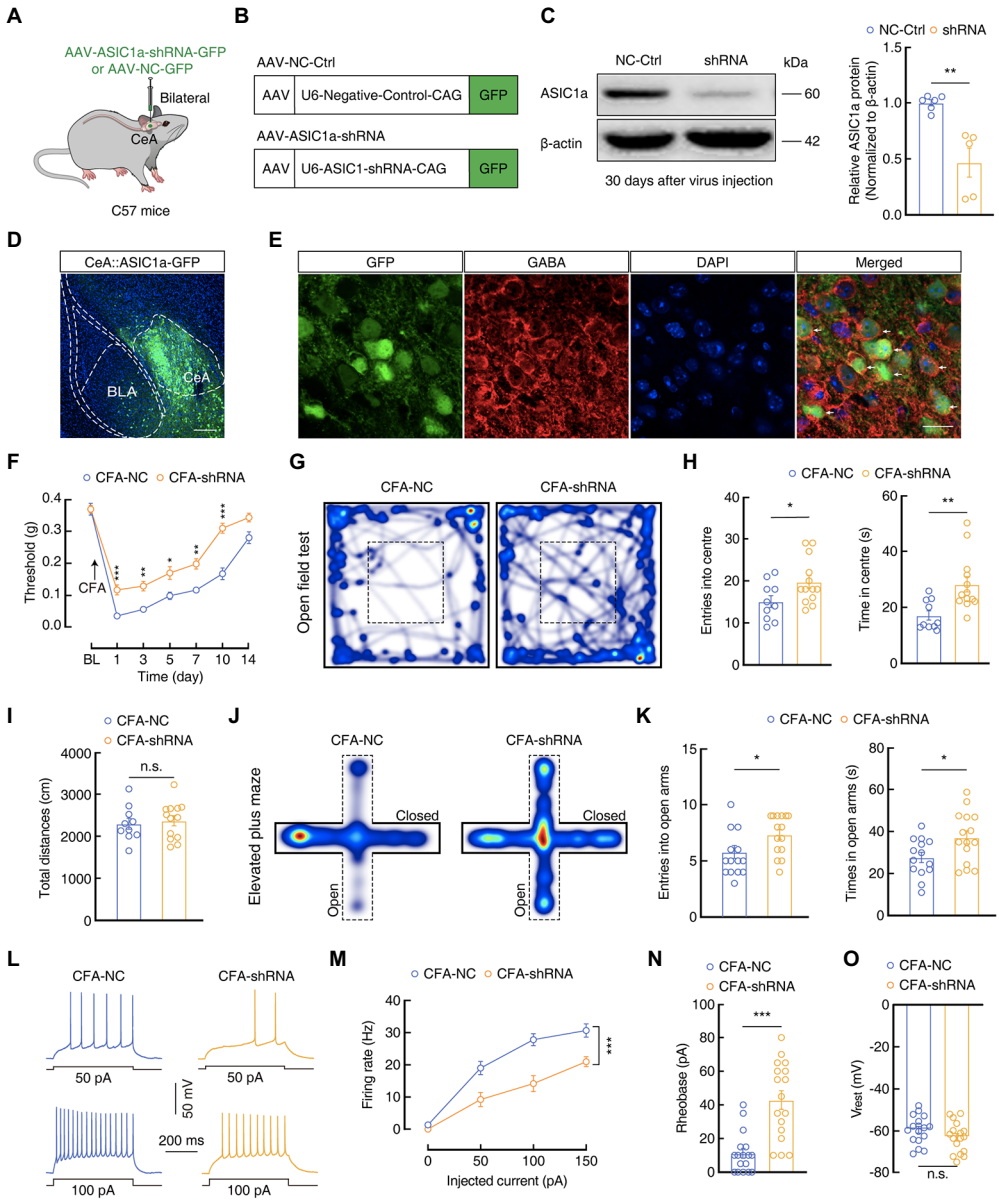
Genetic manipulations of ASIC1a in the CeA influence inflammatory pain hypersensitivity and anxiety-like behaviors. **(A)** Schematic of the genetic knockdown experiment in C57 mice. **(B)** Schematic illustration of the viral vectors for AAV-NC-Ctrl-GFP and AAV-ASIC1a-shRNA-GFP. **(C)** Representative images and quantification of western blots showing that AAV-ASIC1a-shRNA injection reduced ASIC1a expression in the CeA (NC-Ctrl, *n* = 6 mice; shRNA, *n* = 5 mice; *t*_9_ = 4.408, *p* = 0.0017). **(D,E)** Representative images of AAV-ASIC1a-shRNA-GFP expression in the CeA of C57 mice. Scale bar, 200 μm; *GFP*-labeled neurons within the CeA were colocalized with GABA immunofluorescence. Scale bar, 20 μm. **(F)** Genetic knockdown of ASIC1a in the CeA produced analgesic effects (CFA-NC, *n* = 14 mice; CFA-shRNA, *n* = 16 mice; *F*_(1, 28)_ = 40.26, *p* < 0.0001). **(G,J)** Heatmaps showing the locations of CFA-NC mice and CFA-shRNA mice in the open field test (OFT) and elevated plus maze test (EPMT). **(H,I,K)** Genetic knockdown of ASIC1a in the CeA significantly attenuated anxiety-like behaviors (**H**, CFA-NC, *n* = 10 mice; CFA-shRNA, *n* = 13 mice in OFT; entries in center in OFT, *t*_21_ = 2.187, *p* = 0.0402; time in center in OFT, *t*_21_ = 3.226, *p* = 0.004; **I**, total distances in OFT, *t*_21_ = 0.3884, *p* = 0.7017; **K**, CFA-NC, *n* = 14 mice; CFA-shRNA, *n* = 15 mice in EPMT; entries in the open arms in EPMT, *t*_27_ = 2.661, *p* = 0.013; time in the open arms in EPMT, *t*_27_ = 2.392, *p* = 0.024). **(L,M)** Sample traces **(L)** and statistical data **(M)** for action potential firing recorded from GABA^CeA^ in CFA 1D mice treated with AAV-NC-Ctrl or AAV-ASIC1a-shRNA (*n* = 17 cells from four mice per group; F_(1,32)_ = 17.86, *p* = 0.0002). **(N)** Summary of the rheobases of action potentials from CFA 1D mice treated with AAV-NC-Ctrl or AAV-ASIC1a-shRNA (*n* = 17 cells from four mice per group; *t*_32_ = 5.069, *p* < 0.0001). **(O)** The resting membrane potential (V_rest_) showing no difference between CFA-NC mice and CFA-shRNA mice (*n* = 17 cells from four mice per group; *t*_32_ = 1.511, *p* = 0.1406). All data are shown as the mean ± SEM, n.s., not significant; * *p* < 0.05, ** *p* < 0.01 and *** *p* < 0.001. Two-way repeated-measures ANOVA with Bonferroni *post-hoc* analysis for **(F,M)**; unpaired *t*-test for **(C,H,I,K,N,O)**.

To further verify the effect of ASIC1a in GABA^CeA^ neurons, we bilaterally infused a pAAV-U6-ASIC1a-shRNA-CAG-GFP (AAV-ASIC1a-shRNA) or a negative control virus pAAV-U6-negative-control-CAG-GFP (AAV-NC-Ctrl) into the CeA of C57 mice (Figure 7A). Four weeks later, western blotting analysis proved that AAV-ASIC1a-shRNA reduced ASIC1a levels in the CeA compared with AAV-NC-Ctrl (1.00 ± 0.03 *vs* 0.46 ± 0.13; CFA-NC, *n* = 6 mice; CFA-shRNA, *n* = 5 mice; Figures 7B,C). In addition, immunofluorescence staining showed that neurons with a positive green fluorescent protein in the CeA were almost colocalized with a GABA-specific antibody (Figures 7D,E), indicating that the AAV-ASIC1a-shRNA virus-induced knockdown of ASIC1a levels in the CeA was mainly in GABA neurons. Moreover, we found that the genetic knockdown of ASIC1a in the CeA alleviated pain sensitization at 1 to 10 days after CFA injection (Day 1:0.03 ± 0.002 g *vs* 0.12 ± 0.02 g; CFA-NC, *n* = 14 mice; CFA-shRNA, *n* = 16 mice; Figure 7F), which suggested that down-regulation of the ASIC1a gene disrupted the development of inflammatory pain behaviors. Further, genetic downregulation of ASIC1a in the CeA also relieved anxiety-like behaviors (OFT, entries: 15.10 ± 1.43 *vs* 19.77 ± 1.51; time: 17.11 ± 1.66 s *vs* 28.24 ± 2.73 s; CFA-NC, *n* = 10 mice; CFA-shRNA, *n* = 13 mice; EPMT, entries: 5.50 ± 0.52 *vs* 7.33 ± 0.45; time: 27.61 ± 2.42 s *vs* 37.18 ± 3.13 s; CFA-NC, *n* = 14 mice; CFA-shRNA, *n* = 15 mice; Figures 7G–K), and reduced the hyperactivity of GABA^CeA^ neurons (Firing rate: 100 pA, 27.82 ± 1. 87 vs. 14.18 ± 2.49; Rheobase: 11.18 ± 2.93 pA *vs* 42.94 ± 5.54 pA; *n* = 17 cells from four mice per group; V_rest_: −59.43 ± 1.62 mV *vs-63*.03 ± 1.747 mV, *n* = 17 cells from four mice per group; Figures 7L–O) in the CFA-shRNA group compared with those in the CFA-NC group. For Saline 1D mice, we found that there were no significant differences in mechanical pain threshold, anxiety-like behaviors, or GABA^CeA^ neuronal activity between the CFA-shRNA group and CFA-NC group (Supplementary Figure S4). Our collective findings from these disparate experimental manipulations suggest that the downregulation of ASIC1a function in GABA^CeA^ neurons sufficiently and effectively rescued the enhancement of the activity of GABA^CeA^ and CASP in mouse models of acute pain.

## 4. Discussion

In the current study, we demonstrated that the ASIC1a in GABA^CeA^ neurons exerted a regulatory role in pain sensitization and anxiety-like behaviors in an acute pain mouse model. Our results show that the downregulation of ASIC1a function in GABA^CeA^ neurons by either pharmacological inhibition or genetic knockdown significantly alleviated CFA-induced mechanical pain sensitization and anxiety-like behaviors, as well as reduced the hyperactivity of GABA^CeA^ neurons in a mouse model of acute pain. These findings, therefore, indicate that ASIC1a may play an important role in the upregulation of the activity of GABA^CeA^ neurons, which contributes to the development of anxiety-like behaviors under acute pain conditions.

The appropriate treatment of CASP is a major difficulty in this field because its mechanism is not well understood. Traditional analgesics are less effective in patients with CASP; therefore, understanding the mechanism of why patients are far from satisfactory would be a major breakthrough. Norepinephrine reuptake inhibitors and serotonin, as well as benzodiazepines, are typically used to treat patients with pain and anxiety (Bair et al., 2003; Zhang et al., 2016; Gosmann et al., 2021), but approximately one-third of patients do not respond well to the treatment. Given the poor treatment outcomes, exploring promising targets for the treatment of CASP is imperative.

The CeA receives broad excitatory inputs from most brain regions and is involved in regulating different physiological functions (Cai et al., 2018). For example, the role of the amygdala in the process of conditioning fear and mood disorders has been extensively studied (Haubensak et al., 2010). The activity of GABA^CeA^ neurons increases in comorbid pain in depression state; interestingly, inhibition of GABA^CeA^ neurons can alleviate both pain and depressive behaviors in mice (Zhu et al., 2019). In contrast, increasing the activity of the amygdala can reduce the pain threshold in normal rats (Han et al., 2010; Kolber et al., 2010). Consistent with these findings, our results proved that the activity of GABA^CeA^ increased after CFA injection in mice. Chemogenetic inhibition of GABA^CeA^ neurons reduced CFA-induced pain sensitization and anxiety-like behaviors, accompanied with reversal of the hyperactivity of GABA^CeA^ neurons. Hence, we hypothesized that GABA^CeA^ play a key role in CASP in an acute pain mouse model.

The molecular features and morphological localization of GABA^CeA^ neurons are genetically, physiologically, and functionally heterogeneous (Janak and Tye, 2015; Wilson et al., 2019; Li and Sheets, 2020). Different species of GABA^CeA^ neurons, including those expressing adrenocorticotropin-releasing hormone (CRH), protein kinase C delta (PKCδ), somatostatin (SOM), and neurohypophysin (Duvarci and Pare, 2014; Kim et al., 2017; McCullough et al., 2018), may have different roles in pain and anxiety regulation (Wilson et al., 2019; Adke et al., 2021). For example, in a mouse model of the nerve injury, PKCδ^CeA^ neurons were sensitized, leading to nociceptive hyperalgesia, but the excitability of SOM^CeA^ neurons was suppressed, resulting in hypoalgesia (Wilson et al., 2019). This evidence indicates that various types of GABA^CeA^ neurons may play different or even opposite roles in the development of pain and anxiety. In the present study, we take advantage of molecular genetic approaches to dissect the functions of SOM^CeA^ and PKCδ^CeA^ neurons, the majority constituent of the CeA GABAergic neurons, and found that the excitability of SOM^CeA^ and PKC^CeA^ are markedly enhanced in CFA 1D mice. Our findings likely suggest that SOM^CeA^ and PKCδ^CeA^ neurons exert synergistic effects to increase the inhibitory outputs from the CeA in the current CFA-induced inflammatory mouse model.

In addition to processing pain-related information, the CeA is essential for emotional disorders, including addiction, autism, fear, and anxiety (Murray, 2007; Morrison and Salzman, 2010; Johansen et al., 2011; Duvarci and Pare, 2014; Stamatakis et al., 2014). To better understand how the CeA contributes to a wide array of emotional disorders-related behaviors, it will be necessary to identify the functions of heterogeneous neuronal subtypes. A previous study has shown that SOM^CeA^ neurons are activated in response to the conditioned stimulus in fear conditioning in mice (Yu et al., 2016), and chemogenetic inhibition of SOM neurons in the lateral subdivision of CeA (SOM^CeL^) prevented the fear acquisition (Li et al., 2013). In contrast, PKCδ^CeL^ neurons in mice are reported to be hypoactivity in response to the conditioned stimulus following fear conditioning (Ciocchi et al., 2010). Moreover, optogenetic activation of PKCδ^CeA^ neurons produces real-time aversion (Yu et al., 2017) in mice, predicting that endogenous activation of PKCδ^CeA^ neurons after tissue-injury underlies pain-induced aversion (Wilson et al., 2019). Another report showed that optogenetically silencing PKCδ neurons in the lateral subdivision of CeA (CeL) causes a decrease in the anxiety-like behaviors (Botta et al., 2015). Although the excitability of both SOM^CeA^ and PKCδ^CeA^ neurons was increased in the current CFA-induced inflammatory mouse in this study, they may exert different roles in the regulation of emotional disorder-related behaviors, such as anxiety and fear, and the further mechanisms underlying the heterogeneous functions remain to be investigated.

ASIC1a has been reported that it expressed in brain structures that mediate emotion and cognition (including the amygdala, nucleus accumbens, habenula, and periaqueductal grey), suggesting that ASIC1a can affect psychiatric symptoms (Smoller et al., 2014; Gugliandolo et al., 2016; Gonzalez-Inchauspe et al., 2020). For example, ASIC1a knockout mice showed deficits in contextual fear conditioning and unconditioned fear behaviors, such as auditory startle responses and open field center avoidance (Wemmie et al., 2002; Coryell et al., 2008; Taugher et al., 2017; Wang et al., 2018). Notably, ASICs are widely distributed in the cell bodies and sensory terminals of peripheral sensory neurons and are believed to play an important role in pain perception (Wemmie et al., 2006; Fu et al., 2016; Verkest et al., 2018; Wang et al., 2020). Peripheral inflammatory stimulation increased the levels of ASIC1a and ASIC2a in the spinal cord, suggesting that ASIC1a plays a role in central pain sensitization (Duan et al., 2007). In addition, a previous study also revealed that there was no significant variation in ASIC1a subunit mRNA or total ASIC1a protein expression in the basolateral amygdala (BLA) in monoarthritis mice compared to the sham group (Aissouni et al., 2017). Here, we also found that there were no changes of the ASIC1a protein levels of tissues and membrane extractions from the CeA in the CFA 1D mice. Thus, these findings may suggest a complexity of ASIC1a function in peripheral and central nerves systems in the regulation of anxiety-like behaviors in acute pain mice.

However, the mechanisms of ASIC1a action in CASP are still unclear, and it may be that ASICs change neuronal excitability or synaptic plasticity under pain conditions. Previous studies have shown that ASIC1a plays differential roles in synaptic plasticity and transmission in different brain regions (Alvarez de la Rosa et al., 2003). ASIC1a appears to use different downstream signaling pathways to regulate synaptic plasticity (Liu et al., 2016). In the spinal cord, ASIC1a may mediate pain through the BDNF/PI3K/Akt pathway (Duan et al., 2012). Li et al. (2019) have revealed that ASIC1a mediates pain-related synaptic plasticity through protein kinase C lambda (PKCλ) in the ACC. In addition, genetic studies have been used to assess the potential link between ASIC and mood disorders, while these genetic results are encouraging, they are not conclusive (Hettema et al., 2008). Coryell et al. (2009) have suggested that abnormal ASIC function may lead to psychiatric illness, and that targeting ASICs may be beneficial for treatment. In this study, we found that the activity of GABA^CeA^ decreased through genetic knockdown or pharmacological inhibition of ASIC1a. Thus, we hypothesized that ASIC1a produces its effects by altering the activity of neurons. However, the molecular mechanisms of how ASIC1a affects the activity of GABA neurons are still unclear, and further research is warranted.

It has been reported that blockade of ASIC1a can produce potent long-term analgesic effects on pain in rodents. For example, a previous study showed that pharmacologically blocking the activation of ASIC1a by bilaterally injecting PcTx1 into the ACC could alleviate mechanical pain sensitization for up to 7 days in inflammatory pain mice model (Li et al., 2019), which was consistent with our findings in the present study. In addition, a previous study showed that blockade of ASIC1a by both intrathecal and intracerebroventricular injections of PcTx1 can lead to the activation of the endogenous enkephalin pathway in the brain and relieve the thermal and mechanical nociception in neuropathic pain mice model (Mazzuca et al., 2007). As endogenous opioid peptides, enkephalins are released from many brain regions, including the ventrolateral periaqueductal gray (PAG; Williams et al., 1995; Terashvili et al., 2008), and are potent ligands of μ-and δ-receptors and can exert endogenous analgesic effects. Our previous study demonstrated that GABA^CeA^ neurons provided inhibitory projections to glutamatergic neurons in the ventrolateral periaqueductal gray (Glu^PAG^; Yin et al., 2020), which is an important endogenous analgesic region in the brain (Le Merrer et al., 2009). Given these findings, we speculate that the decreased GABA^CeA^ neuronal activity generated by administration of PcTx1 may relieve inhibitory inputs to Glu^PAG^ neurons, thus improving the descending analgesic effects from the PAG in pain mice model. Of course, the potential mechanisms driving this long-lasting effect of ASIC1a on the relieve of pain sensitization and anxiety-like behaviors remain to be further investigated.

In conclusion, our study indicates the critical effect of ASIC1a in GABA^CeA^ in the regulation of pain sensitization and anxiety-like behaviors. As the clinical analgesic drugs currently administered for CASP treatment remain unsatisfactory, our findings suggest ASIC1a channels as potential therapeutic targets in the management of CASP.

## Data availability statement

The original contributions presented in the study are included in the article/Supplementary material, further inquiries can be directed to the corresponding authors.

## Ethics statement

The animal study was reviewed and approved by the University of Science and Technology of China.

## Author contributions

YJ and ZZ conceived and designed the experiments. PS, M-JZ, AL, C-LY, and J-YY carried out the experiments, analyzed the data, prepared figures. RH and YM gave guidance on experimental techniques. PS wrote the manuscript. YJ, WW, and L-SL revised the manuscript. All authors contributed to the article and approved it for publication

## Funding

This work was supported by the National Natural Science Foundation of China (grants 82171218, 81971264, 32271176, 81100558, and 82101301), Youth Innovation Promotion Association CAS, CAS Project for Young Scientists in Basic Research (YSBR-013), CAS Collaborative Innovation Program of Hefei Science Center (2021HSC-CIP013), the Fundamental Research Funds for the Central Universities (WK9100000030), USTC Research Funds of the Double First-Class Initiative (YD9100002018), and the Natural Science Foundation of Anhui Province (2208085J30).

## Conflict of interest

The authors declare that the research was conducted in the absence of any commercial or financial relationships that could be construed as a potential conflict of interest.

## Publisher’s note

All claims expressed in this article are solely those of the authors and do not necessarily represent those of their affiliated organizations, or those of the publisher, the editors and the reviewers. Any product that may be evaluated in this article, or claim that may be made by its manufacturer, is not guaranteed or endorsed by the publisher.
