# Supplementary figures and images for: Acid-sensing ion channel 1a in the central nucleus of the amygdala regulates anxiety-like behaviors in a mouse model of acute pain
Source: Front Mol Neurosci. 2023 Jan 12;15:1006125. doi: 10.3389/fnmol.2022.1006125 (PMC9879607; doi:10.3389/fnmol.2022.1006125)

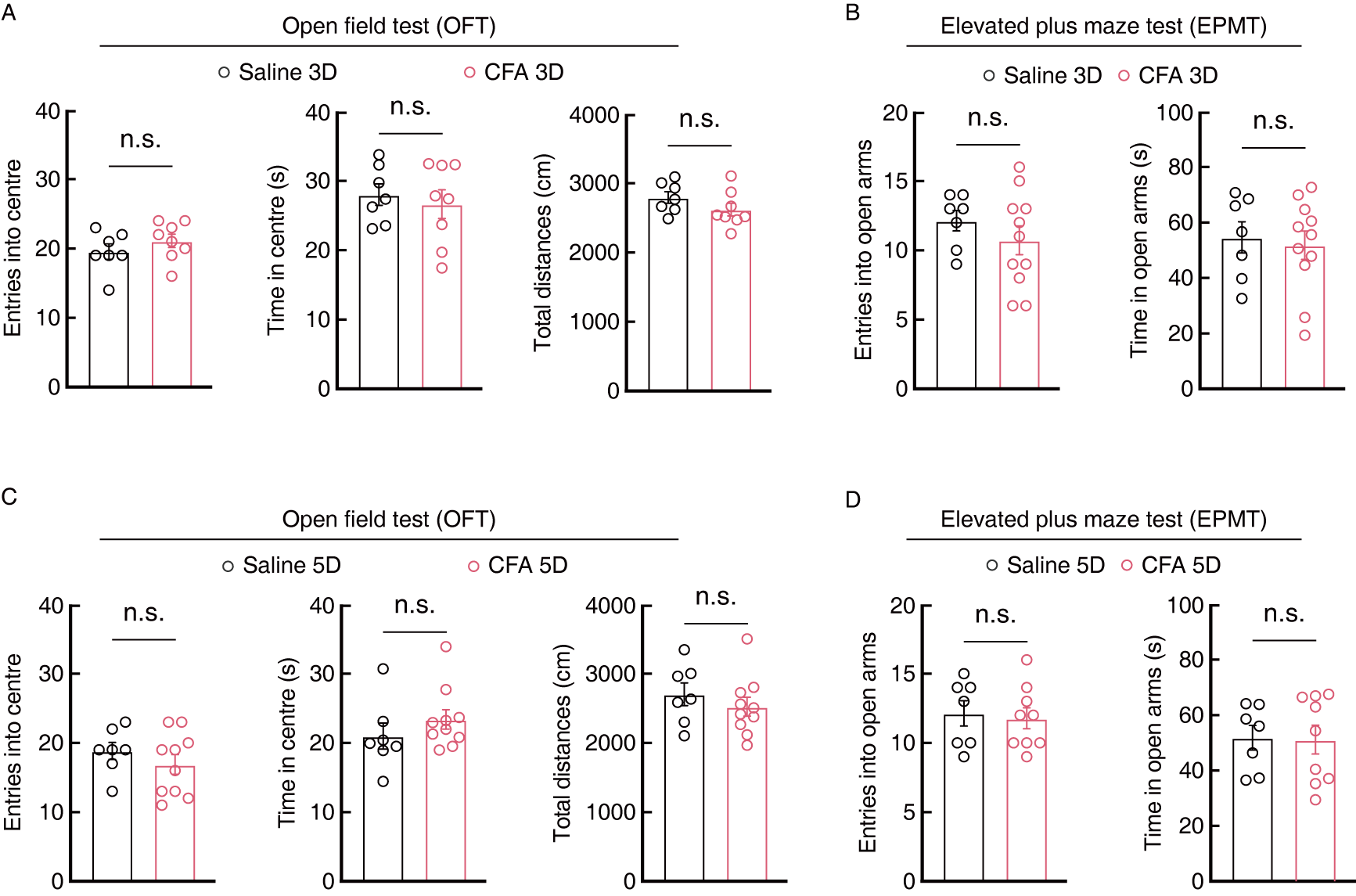

Supplement: Supplementary file 1 [file Image_1.TIF]

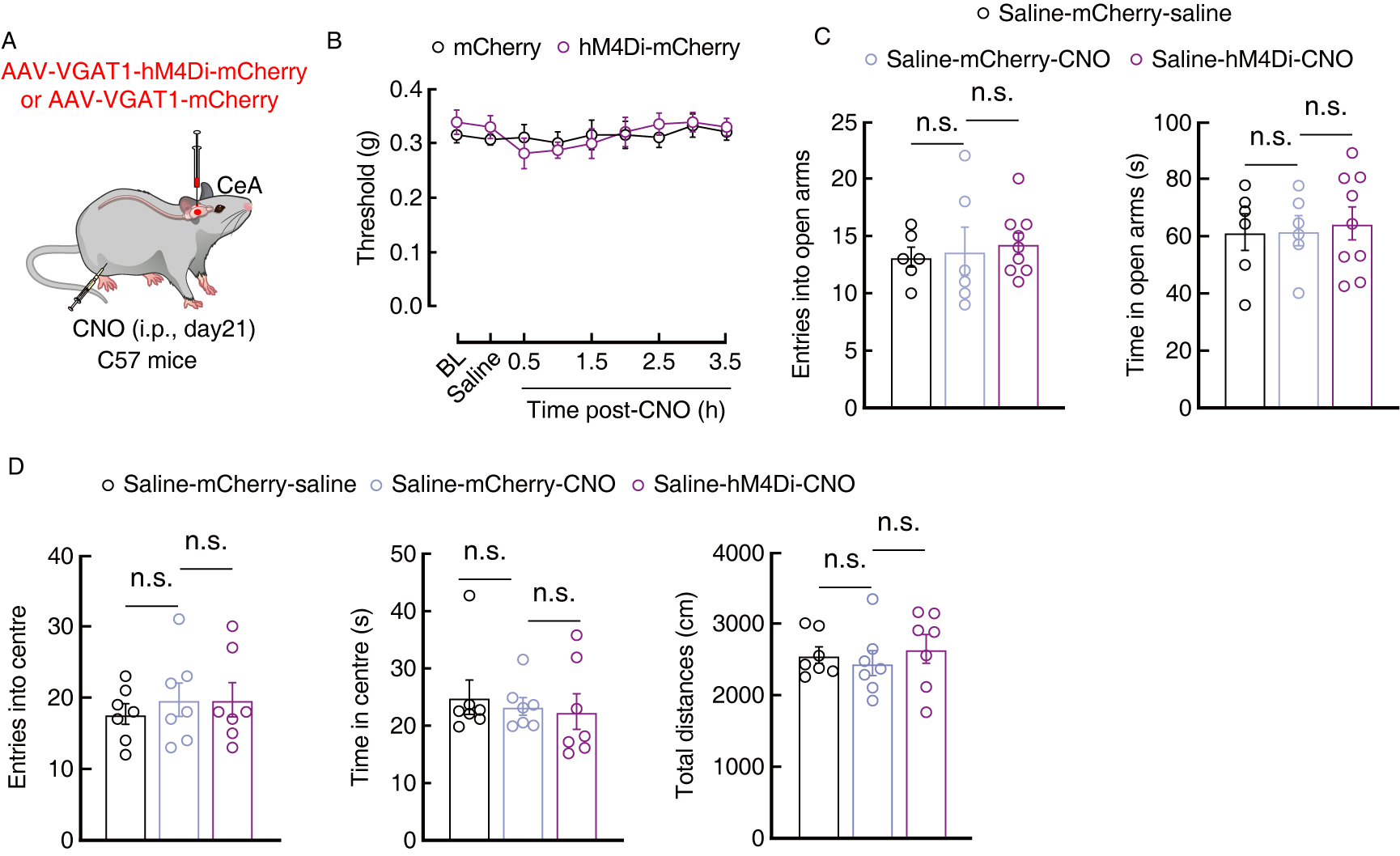

Supplement: Supplementary file 2 [file Image_2.TIF]

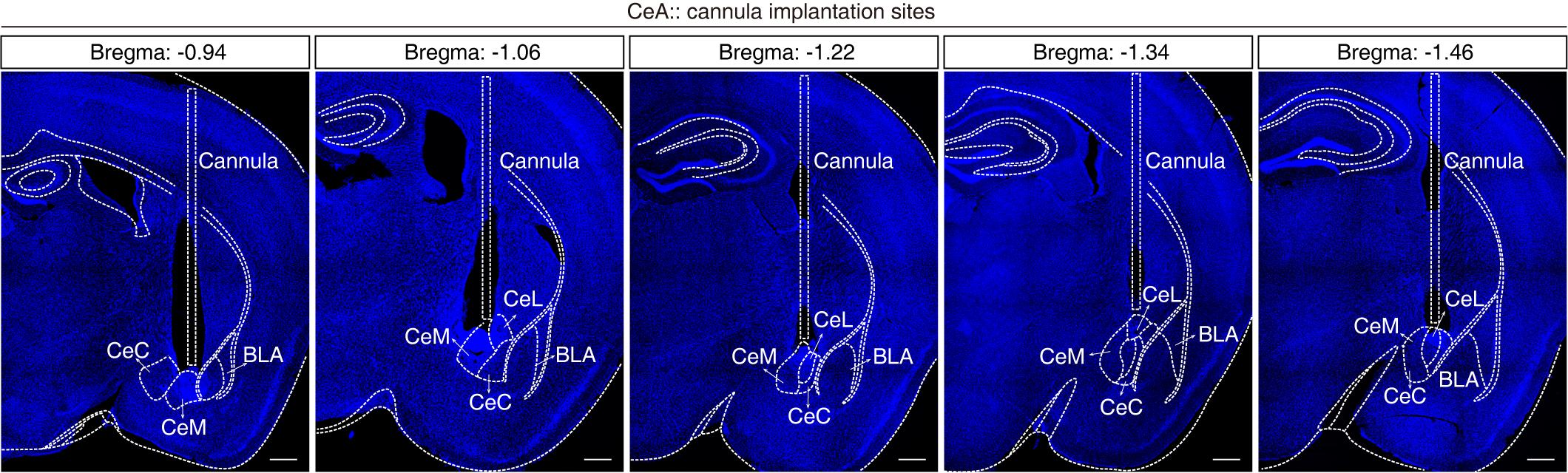

Supplement: Supplementary file 3 [file Image_3.TIF]

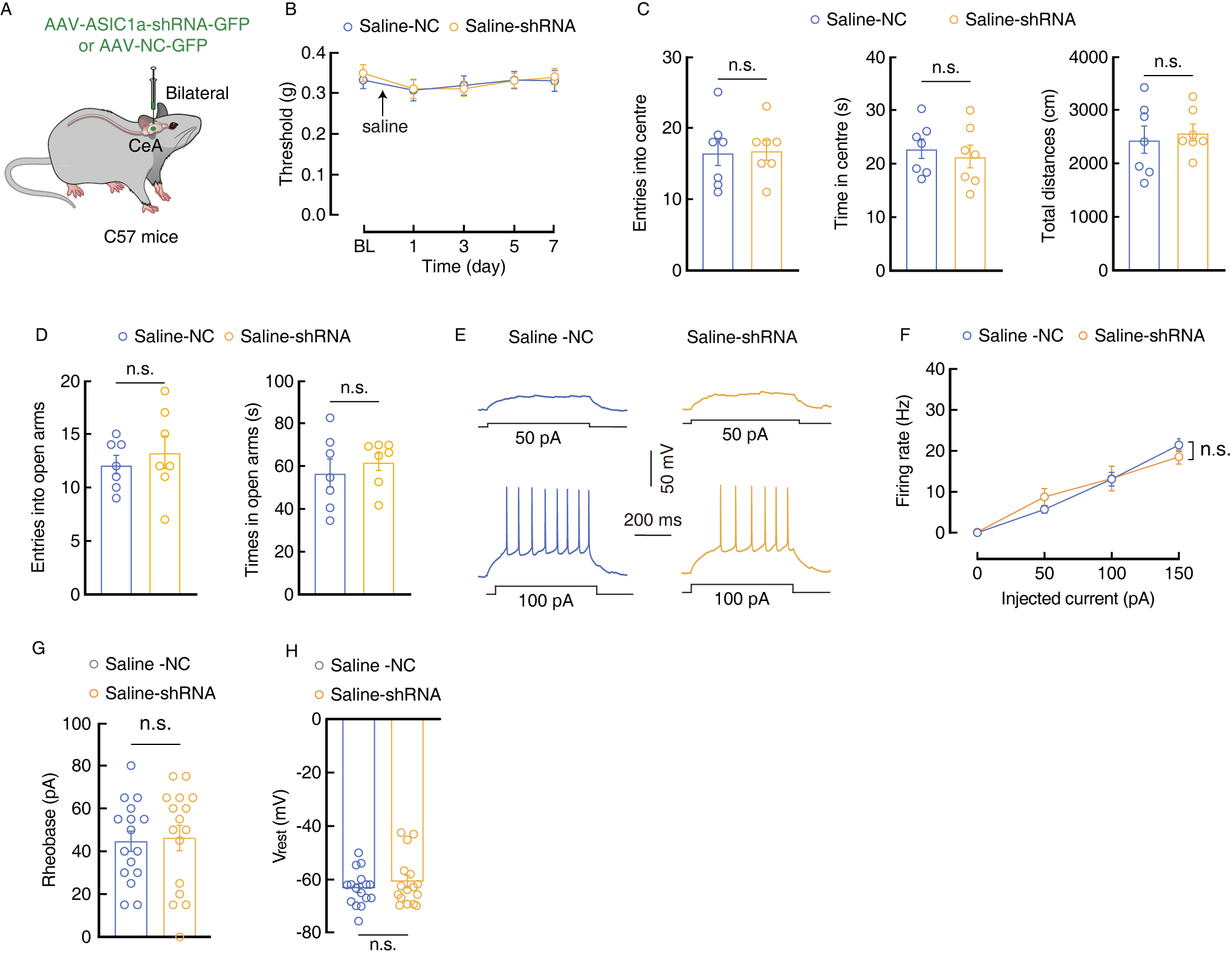

Supplement: Supplementary file 4 [file Image_4.TIF]
